# Synergistic anti-AML effects of the LSD1 inhibitor T-3775440 and the NEDD8-activating enzyme inhibitor pevonedistat via transdifferentiation and DNA rereplication

**DOI:** 10.1038/oncsis.2017.76

**Published:** 2017-09-11

**Authors:** Y Ishikawa, K Nakayama, M Morimoto, A Mizutani, A Nakayama, K Toyoshima, A Hayashi, S Takagi, R Dairiki, H Miyashita, S Matsumoto, K Gamo, T Nomura, K Nakamura

**Affiliations:** 1 Oncology Drug Discovery Unit, Pharmaceutical Research Division, Takeda Pharmaceutical Company Limited, Fujisawa, Japan; 2 Integrated Technology Research Laboratories, Pharmaceutical Research Division, Takeda Pharmaceutical Company Limited, Fujisawa, Japan

## Abstract

Lysine-specific demethylase 1A (LSD1, KDM1A) specifically demethylates di- and monomethylated histones H3K4 and K9, resulting in context-dependent transcriptional repression or activation. We previously identified an irreversible LSD1 inhibitor T-3775440, which exerts antileukemic activities in a subset of acute myeloid leukemia (AML) cell lines by inducing cell transdifferentiation. The NEDD8-activating enzyme inhibitor pevonedistat (MLN4924, TAK-924) is an investigational drug with antiproliferative activities in AML, and is also reported to induce cell differentiation. We therefore tested the combination of these two agents in AML models. The combination treatment resulted in synergistic growth inhibition of AML cells, accompanied by enhanced transdifferentiation of an erythroid leukemia lineage into granulomonocytic-like lineage cells. In addition, pevonedistat-induced rereplication stress during the S phase was greatly augmented by concomitant treatment with T-3775440, as reflected by the increased induction of apoptosis. We further demonstrated that the combination treatment was markedly effective in subcutaneous tumor xenograft models as well as in a disseminated model of AML, leading to tumor eradication or prolonged survival in T-3775440/pevonedistat cotreated mice. Our findings indicate the therapeutic potential of the combination of LSD1 inhibitors and pevonedistat for the treatment of AML.

## Introduction

Acute myeloid leukemia (AML) is a highly aggressive hematological disorder caused by the malignant transformation of hematopoietic stem cells or myeloid progenitor cells, and is also the most common form of adult acute leukemia. Approximately 19,950 new cases are reported annually in the United States and the 5-year survival is reported to be 26%.^1^ Despite advances in the understanding of this disease, the therapeutic strategy has changed little in recent decades. The standard induction chemotherapy comprises 7 days of cytarabine plus 3 days of anthracyclines (7+3 regimen), followed by consolidation of high-dose chemotherapy or stem cell transplantation. Despite intensive therapy, the relapsed/refractory disease rate remains a significant clinical problem. Therefore, novel therapeutic options are urgently needed.

Lysine-specific demethylase 1A (LSD1), the first histone demethylase discovered, specifically demethylates histone H3K4 and H3K9 and serves as a transcriptional corepressor or coactivator, depending on the target gene context.^2, 3^ LSD1 functions as part of a multiprotein complex with corepressor proteins such as CoREST and histone deacetylase 1 (HDAC1).^4, 5^ It is overexpressed in a diverse set of solid tumors as well as hematopoietic malignancies.^6, 7^ Selective small-molecule inhibitors for LSD1 have been reported to show antitumor efficacy in AML.^8, 9, 10^ We found previously that a novel LSD1 inhibitor, T-3775440, inhibits the growth of acute erythroid leukemia and acute megakaryoblastic leukemia cells through enforced transdifferentiation from their original lineages to a myeloid-like lineage.^11^ Given the novel mechanism of action of LSD1 inhibitors, there is a growing interest in potential combinations of LSD1 inhibitors with chemotherapeutics or molecular targeting agents for the treatment of AML. In preclinical models, for example, an LSD1 inhibitor synergistically reduced AML cell viability in combination with cytarabine (Ara-C), a DNA-damaging agent widely used with daunorubicin as standard care for AML.^10^ LSD1 inhibitors also showed synergistic antileukemic effects in combination with an HDAC inhibitor or all-*trans* retinoic acid in AML cell lines.^12, 13^

Pevonedistat is an investigational drug that targets NEDD8-activating enzyme (NAE), leading to the suppression of Cullin-RING E3 ubiquitin ligase (CRL) activity.^14, 15^ Many CRL substrate proteins have pivotal roles in cell cycle, DNA damage repair and differentiation, making NAE a promising anticancer target.^16, 17^ Pevonedistat exhibits significant antitumor activity in multiple preclinical models, including AML.^18^ Notably, single agent clinical activity of pevonedistat has been investigated in AML/myelodysplastic syndrome (MDS).^19^ Efforts have been made to maximize the clinical activity of pevonedistat by combining it with DNA-damaging agents, such as cisplatin, for treating solid tumors.^20, 21^ Pevonedistat triggers the intra-S checkpoint and DNA rereplication, leading to cancer cell death.^22^ In addition to its role in cell cycle machinery, pevonedistat promotes myeloid differentiation of AML cells, leading to antileukemic effects in a xenograft model.^23^ These findings led us to examine the effect of combination treatments of an LSD1 inhibitor and pevonedistat in AML. In this study, we report a synergistic interaction between T-3775440 and pevonedistat in AML cells, highlighting the molecular mechanisms underlying the synergy and robust *in vitro* and *in vivo* antileukemic effects. Our results suggest that LSD1/NAE coinhibition represents a novel therapeutic avenue for the treatment of AML patients with poor prognosis.

## Results

### Combination of T-3775440 and pevonedistat synergistically inhibits AML cell growth

**Figure 1:**
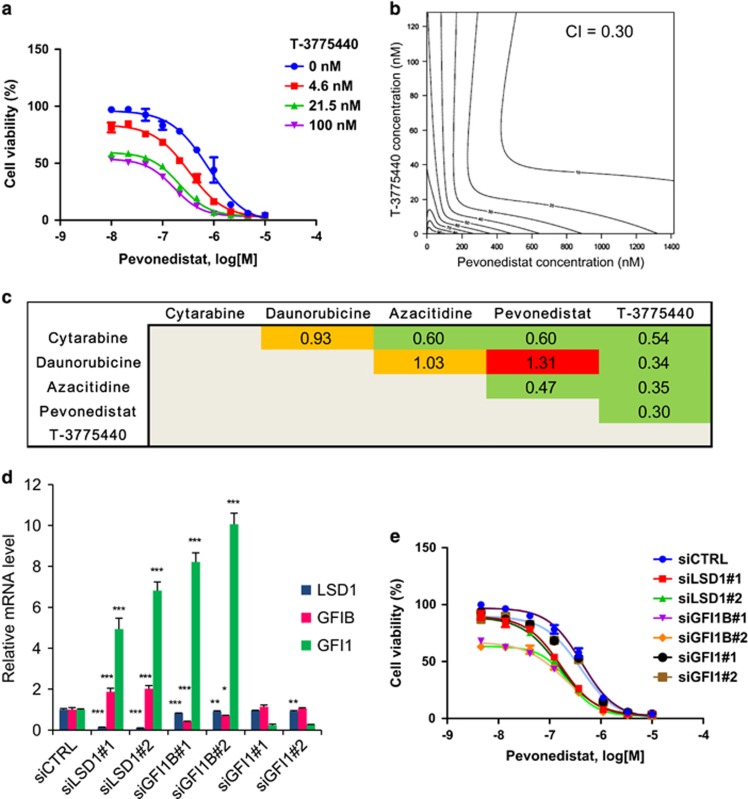
The combination of T-3775440 and pevonedistat shows synergistic growth inhibition of AML cell lines. (**a**–**c**) TF-1a cells were cotreated with T-3775440 and pevonedistat or other anti-AML agents and the effects on cell viability were measured 72 h post treatment using the CellTiter Glo assay. The experiments were conducted in duplicate. (**a**) Representative growth curve of TF-1a cells. (**b**) Isobologram of the cotreatment of TF-1a cells with T-3775440/pevonedistat. (**c**) Values represent the CI for each combination in TF-1a cells. Heat maps are color-coded based on the combination effects: green, synergy (CI values, <0.7); orange, additive (0.7–1.3); red, subadditive (>1.3). (**d**) TF-1a cells were treated with siRNA for 6 h and then replated. At 48 h after the initial treatment, total RNA was purified from the cells and used in quantitative reverse transcription–polymerase chain reaction (qRT–PCR) analyses. The values represent the means of triplicate samples±s.d. Statistical significance was determined using Dunnett's multiple comparison test (*P<0.05, **P<0.01, ***P<0.001). (**e**) TF-1a cells were treated with the indicated siRNA for 6 h and replated as in (**d**). After overnight incubation, cells were treated with pevonedistat for 72 h. Dose–response curve of cells treated with pevonedistat and siRNA is shown (*n*=3).

To analyze the interaction between T-3775440 and pevonedistat in AML cell proliferation, we performed *in vitro* combination studies in a series of AML cell lines. As shown in Table 1 and Supplementary Figure 1, synergistic effects were observed in seven cell lines out of 15 and additive effects were observed in another seven cell lines, suggesting that this combination has a broad anti-AML spectrum. In contrast, T-3775440 had little effect on pevonedistat-mediated growth inhibition of CCRF-CEM and MOLT-3 (acute lymphoblastic leukemia cell lines), RPMI8226 and KMS28BM (multiple myeloma cell lines) or HepG2 (a hepatocellular carcinoma cell line), suggesting that the combination effects were specific for AML cells (Supplementary Figure 2). Since the growth inhibition curve and isobologram indicated a clear synergism in TF-1a erythroloid leukemic cells (Figures 1a and b and Supplementary Table 1) and cytarabine-resistant TF-1a/Ara-C cells (Table 1 and Supplementary Figures 1a and 3), we also evaluated the combination effects of T-3775440 with cytarabine, daunorubicine and azacitidine, which are used for the treatment of AML and/or MDS, in TF-1a cells (Figure 1c and Supplementary Figure 4). T-3775440 exhibited synergistic effects with all agents tested. Among them, pevonedistat exhibited the greatest synergism in combination with T-3775440 in TF-1a cells (FAB-M6) as well as in Kasumi-1 cells (FAB-M2) with a combination index (CI) of 0.30 (Figures 1b and c) and 0.45 (Supplementary Figure 5), respectively.

### GFI1B inhibition by T-3775440 is involved in the combination effects with pevonedistat in TF-1a cells

LSD1 inhibits lineage-specific gene expression by forming transcription repressive complexes with several transcription factors, including CoREST and GFI1B.^5^ The interaction between LSD1 and GFI1B is reported to be responsible for erythroid and megakaryocytic lineage specification.^5, 24^ We recently reported that the antileukemic activity of T-3775440 is mediated by its ability to disrupt the LSD1-GFI1B interaction in GFI1B-expressing AML cells, which leads to derepression of myeloid lineage genes and subsequent cell transdifferentiation.^11^ To test whether the combination effect of T-3775440 and pevonedistat was dependent on the disruption of the LSD1-GFI1B axis, we perturbed LSD1 and GFI1B expression using small interfering RNA (siRNA) in the presence of pevonedistat in GFI1B-expressing TF-1a cells (Figure 1d). LSD1 or GFI1B knockdown derepressed the expression of GFI1, a target gene of the LSD1-GFI1B transcription repressive complex, at a level equal to the effect of T-3775440 (Figure 1d and Supplementary Figure 6)^25^ and significantly lowered the half-maximal effective concentration value of pevonedistat compared with that of the control (Figure 1e and Supplementary Table 2). In contrast, knockdown of GFI1 did not alter the effect of pevonedistat on the viability of TF-1a cells. These results suggest that inhibition of LSD1-GFI1B by T-3775440 is involved in its synergistic interaction with pevonedistat.

### T-3775440 enhances rereplication stress induced by pevonedistat, leading to apoptosis

**Figure 2:**
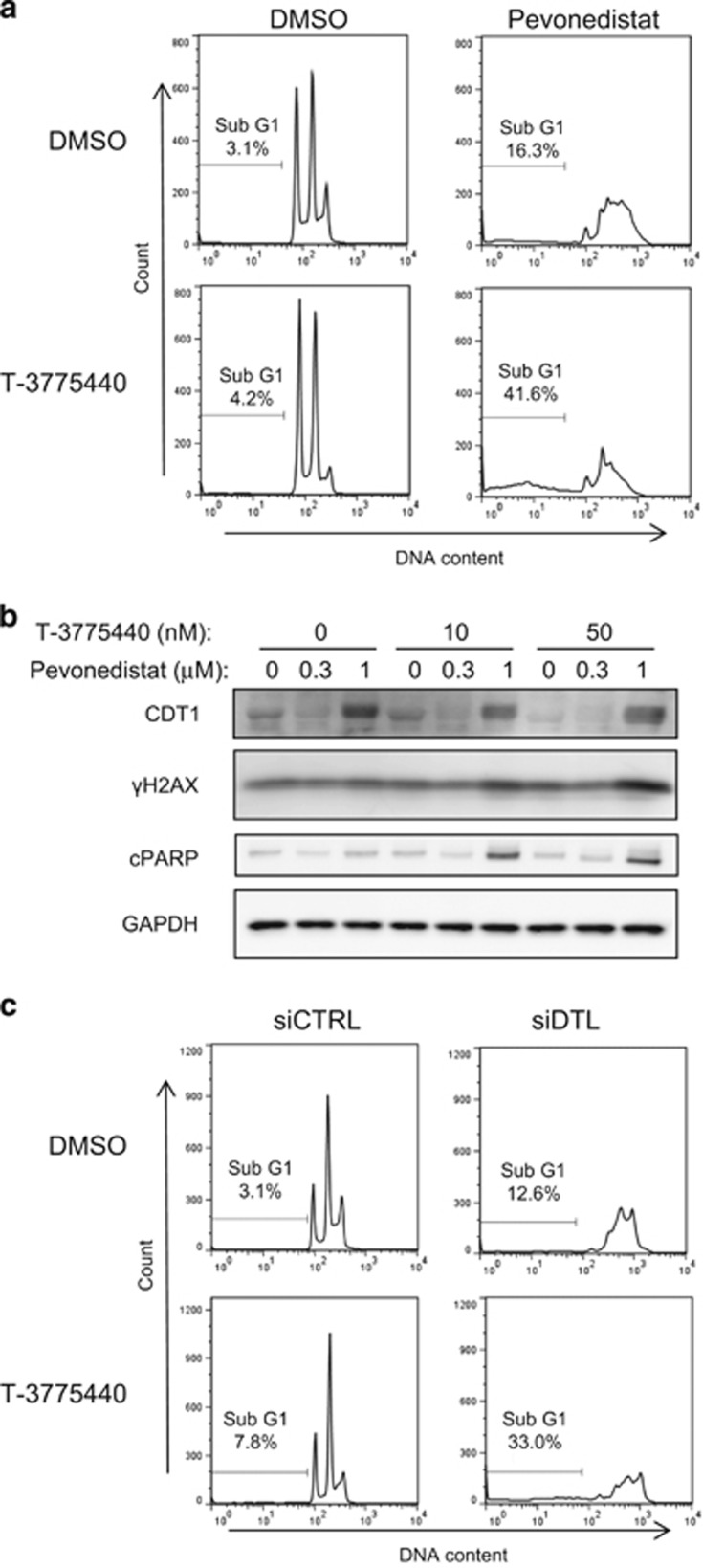
T-3775440 enhances apoptotic cell death under pevonedistat- or DTL depletion-induced replication stress. (**a**) TF-1a cells were treated with vehicle, 50 nm T-3775440, 1000 nm pevonedistat, or T-3775440 and pevonedistat for 48 h. DNA content was determined by flow cytometry. Note that this cell line grows as a stable population of cells with *n*, 2*n* and 4*n* nuclear complements. Percentages of cells in the sub-G1 population are indicated. (**b**) Increased DNA damage and apoptosis after T-3775440 and pevonedistat treatment. TF-1a cells were treated with drugs or dimethyl sulfoxide (DMSO) control (as indicated) for 48 h. Immunoblotting analysis was performed to determine the expression levels of γH2AX and cleaved PARP. Glyceraldehyde 3-phosphate dehydrogenase (GAPDH) was used as a protein-loading control. (**c**) TF-1a cells were transfected with siRNA targeting DTL or control siRNA and incubated for 4 h. Next, cells were replated in the presence or absence of 50 nm T-3775440 for 48 h. Cell cycle analysis was performed using flow cytometry.

Pevonedistat is known to induce DNA rereplication and DNA damage in cancer cells.^26^ Hence, we analyzed cell cycle profiles to determine whether T-3775440 affected the rereplication phenotype induced by pevonedistat treatment. T-3775440 treatment alone moderately increased the number of cells in the sub-G1 fraction, whereas pevonedistat treatment caused dysregulation of the cell cycle progression triggered by DNA rereplication (Figure 2a). The combination of these two agents significantly increased the cell population in the sub-G1 fraction, indicating potentiated apoptotic cell death (Figure 2a). To confirm that cell death was via apoptosis due to DNA damage, we performed western blot analyses. As a single agent, neither T-3775440 nor pevonedistat affected the expression levels of γH2AX and cleaved PARP, markers for double-strand DNA damage and apoptosis, respectively (Figure 2b). In contrast, cotreatment with T-3775440 and pevonedistat significantly increased the signal intensity of γH2AX as well as the cleaved form of PARP (Figure 2b). Apoptotic cell death induced by the combination was also confirmed by the amount of cleaved caspase-3 and caspase-3/7 activities (Supplementary Figures 7a and b), while no clear additive or synergistic effects on proteins involved in the DNA damage response pathway, such as phospho-MCM2, FANCD2, phosho-Chk1 and Chk2, were observed with the cotreatment (Supplementary Figure 7c). It has been reported that pevonedistat induces rereplication via inhibition of the ubiquitin ligase CUL4-DDB1^DTL^ and subsequent CDT1 accumulation, and that knockdown of DTL mimics the S-phase effect of pevonedistat.^26^ Indeed, pevonedistat induced accumulation of CDT1 (Figure 2b) as well as other Cullin-RING ligase substrates p27 (Supplementary Figure 7c) and NRF2 (data not shown). Therefore, we tested the effect of cotreatment with T-3775440 and DTL siRNA, which resulted in significant apoptotic cell death compared with perturbation alone, mimicking the synergistic apoptosis-inducing effect of T-3775440/pevonedistat combined treatment (Figure 2c, Supplementary Figure 8 and Supplementary Table 3). These results suggest that AML cells under rereplication stress are highly vulnerable to T-3775440 treatment.

### Cotreatment with T-3775440/pevonedistat cooperatively induces transdifferentiation of erythroid leukemia cells

**Figure 3:**
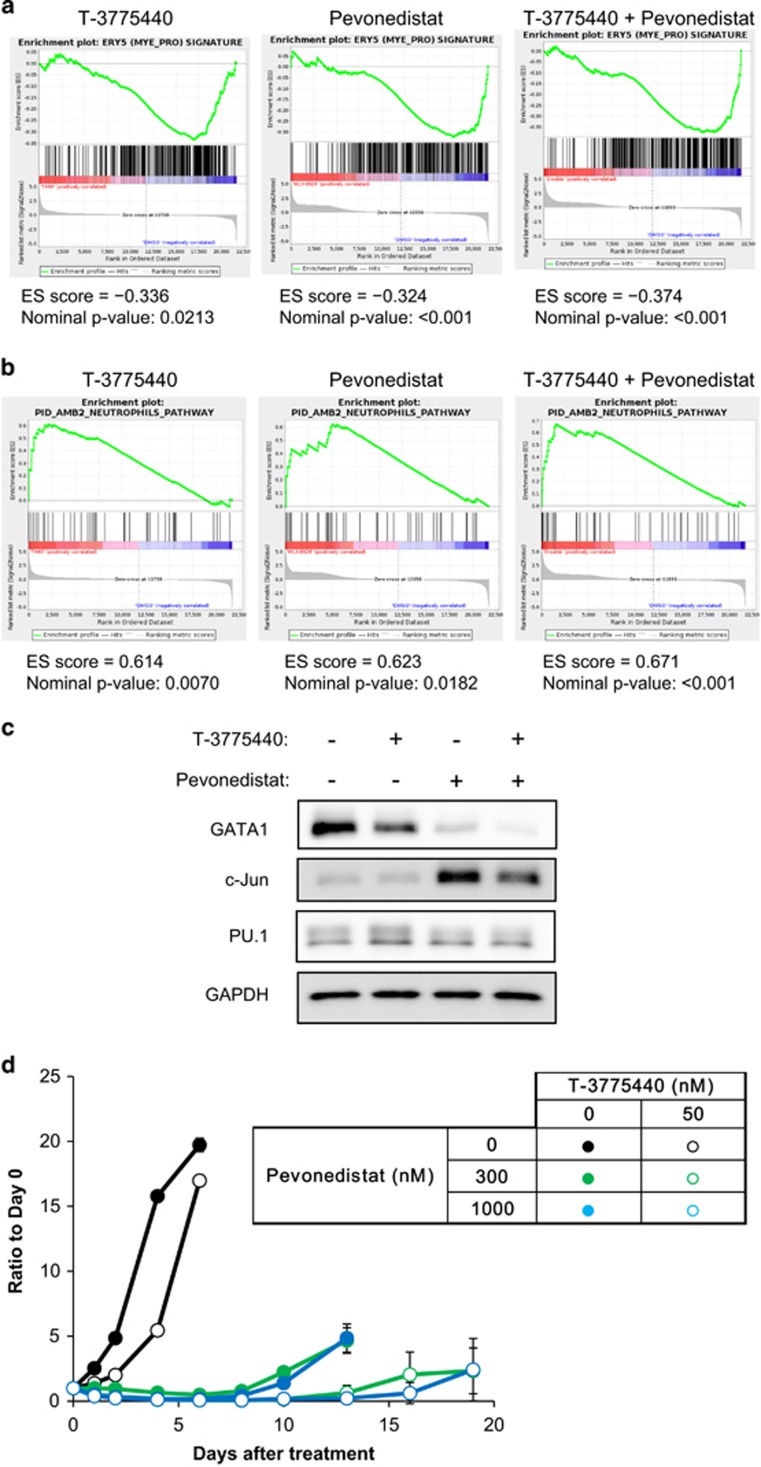
Cotreatment with T-3775440/pevonedistat results in AML cell transdifferentiation and durable growth suppression. (**a**, **b**) TF-1a cells were treated with vehicle, 50 nm T-3775440, 1000 nm pevonedistat or T-3775440 and pevonedistat in combination for 24 h. Total RNA was used for microarray analysis. Gene set enrichment plots demonstrate the downregulation of erythroid signature genes (**a**) and upregulation of neutrophil signature genes (**b**). (**c**) TF-1a cells were treated with T-3775440 and pevonedistat as indicated. Whole-cell lysates were prepared and subjected to immunoblotting analysis. Glyceraldehyde 3-phosphate dehydrogenase (GAPDH) was used as a loading control. (**d**) Cell proliferation assay after washout of compounds. TF-1a cells were treated with the indicated drugs alone or in combination for 72 h. Cells were then replated in the absence of the compounds and proliferation rates were determined using CellTiter Glo assay (*n*=3).

We previously reported that T-3775440 leads to differentiation of AML cells and thereby induces cell growth arrest and apoptosis.^11^ Pevonedistat induces not only DNA rereplication-mediated genotoxic stress but also triggers the differentiation of AML cells.^23^ We therefore examined how cotreatment with these two agents affects transcriptional networks that regulate lineage specificity in TF-1a erythroid leukemia cells. Microarray and gene set enrichment analysis revealed that treatment with T-3775440 or pevonedistat alone downregulated erythroid cell gene expression but upregulated neutrophilic cell gene expression (Figures 3a and b). Cotreatment with these two agents further augmented the degree to which the erythroid and neutrophilic gene signatures were depleted and enriched, respectively, as evidenced by the greater values of negative and positive enrichment scores. Consistent with the results from the gene set enrichment analysis, the expression of several representative erythroid and neutrophil marker genes was more significantly downregulated and upregulated, respectively, by the combination treatment than by treatment with either agent alone (Supplementary Figures 9a and b). These results suggest that this combination cooperatively promoted transdifferentiation in the same direction, from the erythroid lineage to the myeloid-like lineage.

GATA1 is a master transcription factor responsible for erythroid lineage maintenance and commitment, while PU.1 is the counterpart for myeloid lineage.^27^ Erythroid and myeloid lineage commitment is regulated by the balance in activities of these two transcription factors. Thus, we examined the expression levels of these factors in TF-1a cells following treatment with T-3775440 alone, pevonedistat alone or the combination of both. The combination treatment decreased GATA1 levels to a greater extent than did either agent alone in TF-1a cells and in MOLM-16, a megakaryoblastic leukemia cell line (Figure 3c and Supplementary Figure 9c). It also decreased KLF1 levels, which is a direct target of GATA1 in both cell lines (Supplementary Figure 9c). In contrast, the effects of the combination as well as each single agent on PU.1 levels were modest. We next examined the protein expression level of c-Jun, a transcription cofactor known to enhance the transcriptional activity of PU.1. Consistent with previous reports that pevonedistat increases c-Jun by inhibiting SCF-type ubiquitin ligase Fbxw7-mediated degradation,^28, 29^ pevonedistat treatment increased c-Jun protein levels in TF-1a cells (Figure 3c). The PU.1 target genes CCAAT/enhancer binding protein α (*CEBPA*) and *CD86* were additively upregulated by cotreatment with T-3775440 and pevonedistat (Supplementary Figures 9d and e). These results suggest that T-3775440 and pevonedistat cooperatively promote cell differentiation by shifting the balance from GATA1 to PU.1/c-Jun in TF-1a cells.

To assess the durability of cotreatment-induced growth inhibition, we performed a washout study in TF-1a cells. After exposure to each agent alone or to the combination treatment, cells were replated into media free from either agent (Figure 3d). Pretreatment with T-3775440 alone did not significantly delay cell regrowth after washout, reflecting the cytostatic effect of T-3775440 at this concentration. Pretreatment with pevonedistat alone caused relatively durable cell growth inhibition up to 7 days after the washout. Cotreatment, however, demonstrated even more prolonged antiproliferative effects, with regrowth of cells not observed for almost 2 weeks.

### Coadministration of T-3775440/pevonedistat exhibits significant antitumor activity in subcutaneous AML xenograft models

**Figure 4:**
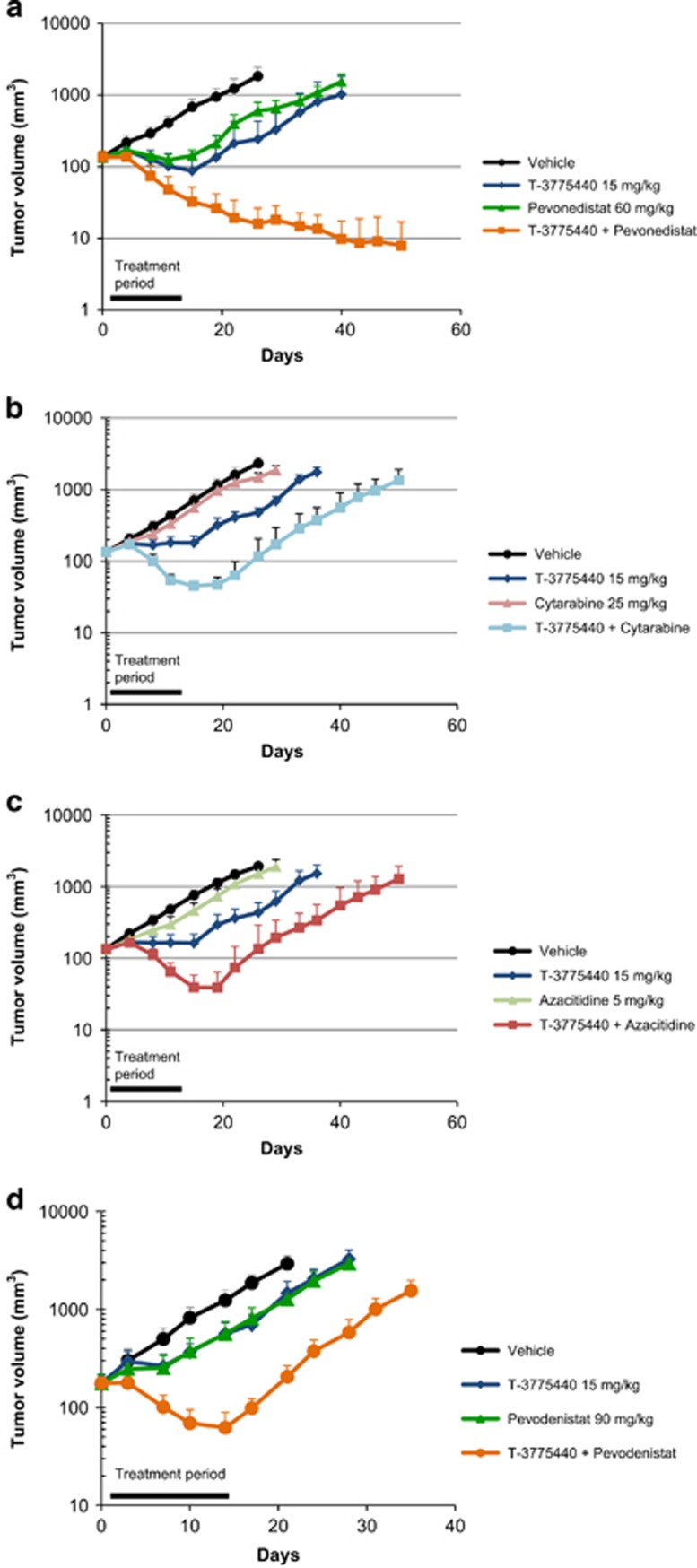
The T-3775440/pevonedistat combination exhibits significant anti-AML effects in subcutaneous xenograft models. (**a**–**c**) Antitumor effects of T-3775440 in combination with pevonedistat (**a**), cytarabine (**b**) or azacitidine (**c**) were examined in TF-1a tumor subcutaneous models. Mice were subcutaneously (s.c.) inoculated in the flank with AML cells. Animals received T-3775440 once daily orally (p.o.) on a 5 days on/2 days off schedule, pevonedistat three times weekly (on days 1, 3 and 5, s.c.), cytarabine three times weekly (on days 1, 3 and 5, intraperitoneally (i.p.)), or azacitidine two times weekly (on days 1 and 4, s.c.). The values represent mean tumor volumes±s.e.m. (*n*=5). (**d**) Antitumor effects of the T-3775440/pevonedistat combination were examined in a MOLM-16 model with the same dosing schedule as in (**a**).

*In vitro* combination studies often overestimate the effect of combination treatment, as they do not consider potential dose reduction to mitigate adverse effects caused by coadministration. Therefore, we used AML xenograft mouse models to examine whether coadministration of T-3775440/pevonedistat produced *in vivo* antitumor effects as observed *in vitro*. The combination of T-3775440 (15–20 mg/kg, orally, on a 5 days on/2 days off schedule) and pevonedistat (60–90 mg/kg, subcutaneously, three times per week on days 1, 3 and 5) was tolerated for 2 weeks (Figure 4a and Supplementary Figures 10a and b). In a TF-1a subcutaneous tumor xenograft model, although treatment with each single agent exhibited a significant antitumor effect, tumors regrew shortly after cessation of treatment. The combination treatment, however, showed more significant antitumor effects during and even after the treatment period. Two mice out of six achieved complete tumor eradication, and all mice had no tumor recurrence throughout an extended observation period (until day 50). Such significant combination effects were also observed in mice that had received only a single cycle of coadministration, which consisted of 5 days of T-3775440 (20 mg/kg, days 1–5) and 2 days of pevonedistat (60 mg/kg, days 1 and 4) (Supplementary Figures 11a and b). We further compared the potential of pevonedistat as a combination partner with T-3775440 to that of cytarabine or azacitidine in the same xenograft models (Figures 4b and c). Although combinations with cytarabine or azacitidine resulted in significant tumor regression during the dosing period in the TF-1a models, neither of them achieved tumor eradication at the maximum-tolerated doses (Supplementary Figures 10c–g). We also examined the combination effect in a model of MOLM-16, a megakaryocytic leukemia cell line, where administration of each agent alone led to only modest tumor growth suppression, but where the combination resulted in sustained tumor regression during the dosing period (Figure 4d and Supplementary Figure 10h).

### Coadministration of T-3775440/pevonedistat reduces tumor burden and improves mouse survival in an erythroid leukemia dissemination model

**Figure 5:**
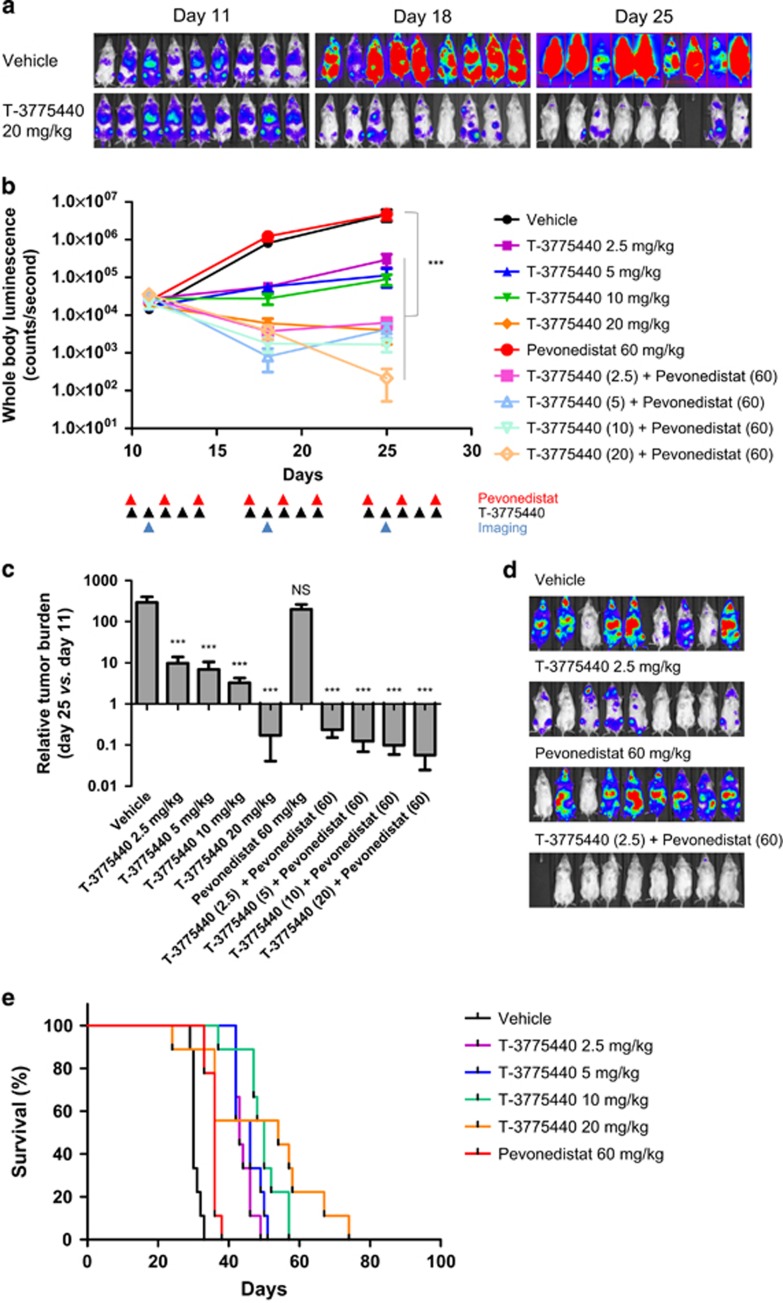

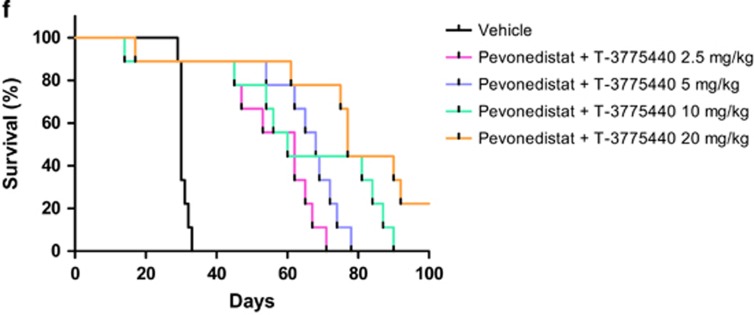
Coadministration of T-3775440/pevonedistat reduced leukemic burden and prolonged animal survival in an AML disseminated model. (**a**–**f**) Mice were inoculated via the tail vein with luciferase-labeled TF-1a cells (day 0). Mice were randomized into groups (*n*=9 per group) and treatment was initiated 10 days after cell injection (day 10). T-3775440 was administrated once daily (p.o.), and pevonedistat three times per week (days 1, 3 and 5, s.c.) according to the indicated dose and schedule. Tumor growth was monitored using an *in vivo* imaging system at days 11, 18 and 25. (**a**) Luminescence images of TF-1a-luc cell-bearing mice that received 20 mg/kg T-3775440 or vehicle are shown. (**b**) Whole-body luminescence of each treatment group is shown at the indicated time points. All values are means, and bars represent the s.e.m. (**c**) Bar charts showing relative tumor burden of each treatment group compared with that at days 11 and day 25. Means and s.e.m. are shown. Statistical significance was determined using Dunnett's multiple comparison test (***P<0.001). (**d**) Representative luminescence images (day 25). (**e**, **f**) Kaplan–Meier analysis was conducted to compare survival curves between vehicle-treated mice and T-3775440, MLN4924 or combination-treated mice. Single agent groups (**e**) and combination groups (**f**) are shown separately with the control group.

To extend our findings in subcutaneously implanted AML xenograft models, the combination effects of T-3775440/pevonedistat were further evaluated in a mouse dissemination model using TF-1a-luc cells, in which leukemic cell growth was monitored noninvasively via emitted bioluminescence. As shown in Figure 5a, tumor cells were disseminated into various organs, including the bone marrow and spleen, as early as 11 days after cell inoculation (day 11), and they proliferated over the monitoring period in vehicle-treated mice (days 11–25). Whole-body luminescence increased ~300-fold during this time period (Figure 5b). T-3775440 significantly delayed tumor outgrowth in a dose-dependent manner over a dose range of 2.5–10 mg/kg, and even reduced tumor burden at 20 mg/kg (Figures 5a–c). Although pevonedistat itself showed little effect on tumor burden in this model, coadministration of T-3775440/pevonedistat led to a reduction in tumor burden, even when T-3775440 was combined at a low dose of 2.5 mg/kg (Figures 5b–d and Supplementary Figures 12a and b). In parallel with the tumor burden changes, T-3775440 treatment significantly prolonged mouse survival compared with vehicle treatment (Figure 5e and Supplementary Table 4). The combination of T-3775440/pevonedistat exhibited more significant prolongation of life than either agent used alone (Figure 5f and Supplementary Table 4). Through extended time periods, 2 out of 10 mice that had received 20 mg/kg of T-3775440 and 60 mg/kg of pevonedistat had no detectable signal of tumor burden at day 133, suggesting that these mice achieved complete remission (Supplementary Figure 12c). The drug treatment was generally tolerated, although one mouse out of nine died during the treatment period in the T-3775440 monotherapy group (20 mg/kg) and the T-3775440/pevonedistat combination groups, possibly due to hemorrhage following multiple injections of luciferin and/or pevonedistat in the context of T-3775440-induced thrombocytopenia. Despite this adverse effect observed in the model, the combination of T-3775440/pevonedistat exerted significant antileukemic effects that led to overall improved mouse survival.

## Discussion

In this study, we demonstrated a synergistic interaction between the LSD1 inhibitor T-3775440 and the NAE inhibitor pevonedistat in various AML models. Cotreatment with these agents significantly suppressed AML cell growth *in vitro* and *in vivo*. Notably, intensive but short-term treatment with T-3775440/pevonedistat resulted in tumor eradication in subcutaneous xenograft models and prolonged survival in a cell-disseminated model of TF-1a erythroid leukemia. Some mice achieved long-term remission and a potential cure following the treatment. The combination of T-3775440 with pevonedistat showed superior activity to combinations with conventional chemotherapeutics such as cytarabine and daunorubicine. These data suggest that a T-3775440/pevonedistat combination regimen represents a novel strategy to treat resistant/refractory AML, beyond conventional cytarabine/anthracyclines ‘7+3’ induction chemotherapy. However, the clinical relevance of the anti-AML effects of the combination needs to be further validated in studies that, for instance, use patient-derived primary AML cells.

The synergistic interaction between T-3775440 and pevonedistat appeared to be most promising in AML cells, despite the fact that the target molecules, LSD1 and NAE, are widely expressed in a range of cancer types. This may reflect the selective activity of the LSD1 inhibitor against AML. In particular, the acute erythroid leukemia cell line TF-1a was highly sensitive to the combination treatment both *in vitro* and *in vivo*. Erythroid leukemia cells express high levels of GFI1B protein, a SNAG domain-containing protein, which is involved in the lineage-specific transcription program through interactions with LSD1. We have shown previously that T-3775440 produces antileukemia effects by targeting a critical interaction between LSD1 and GFI1B transcription repressor in erythroid and megakaryoblastic leukemia cells.^11^ In the present study, GFI1B knockdown potentiated the antileukemia effects of pevonedistat, mimicking the synergistic interaction between T-3775440 and pevonedistat. The lack of a synergistic interaction in the combination in non-AML cells can be explained by the low GFI1B expression in these cell types, which suggests few overlapping nonhematologic toxicities, such as hepatotoxicity,^30^ and a wide therapeutic window for the combination. Thrombocytopenia appeared to be a dose-limiting toxicity of the combination in our preclinical models, although we believe that platelet transfusion would be a feasible approach to manage this adverse effect in clinical settings.

Cotreatment with T-3775440/pevonedistat significantly promoted the transdifferentiation of erythroid leukemia cells. This effect is most likely dependent on the ability of each agent to induce transdifferentiation in the same direction as the megakaryocytic–erythroid to granulocytic–monocytic lineage.^11, 23^ Of note, the T-3775440/pevonedistat combination cooperatively decreased the expression of GATA1, a lineage-restricted transcription factor of erythroid and megakaryocytic cells.^31^ GATA1 physically interacts with and inhibits the activity of the PU.1 transcription factor, a central regulator of myeloid differentiation, in a dose-dependent manner.^32, 33, 34^ In contrast to a previous report wherein pevonedistat significantly increased PU.1 protein levels in MV-4–11 cells,^23^ each agent alone and the combination of T-3775440/pevonedistat only modestly affected PU.1 levels in TF-1a cells. Instead, pevonedistat treatment increased protein expression of c-JUN, a well-known substrate of SCF (SKP1, Cullin and F-box protein) E3 ubiquitin ligase.^28, 35^ c-JUN cooperates with PU.1 and relieves GATA1-mediated repression of a myeloid transcription program.^36, 37^ Indeed, cotreatment with T-3775440/pevonedistat led to significant expression of PU.1-dependent CEBPA and CD86 in TF-1a cells. These data suggest that these key transcription factors, which function in early myeloid linage selection, execute AML cell transdifferentiation induced by the T-3775440/pevonedistat combination.

Consistent with previous studies,^22, 26^ pevonedistat elicited DNA rereplication by stabilizing CDT1 in the S phase, leading to cell apoptosis. We demonstrated that this DNA rereplication-induced cell death was significantly augmented by T-3775440, not only in pevonedistat-treated cells but also in DTL-depleted cells, suggesting that AML cells under rereplication stress are highly susceptible to T-3775440 treatment. Mosammaparast *et al.*^38^ reported that LSD1 was recruited to sites of DNA damage, preferentially in late S/G2 phase, and promoted ubiquitylation of H2A/H2AX, thus enabling a full DNA damage response.^38^ Cotreatment with T-3775440/pevonedistat increased DNA double-strand breakage, as evidenced by γH2AX expression. These results suggest that LSD1 inhibition sensitizes cells to pevonedistat treatment by disabling the DNA damage response, although the exact mechanism has yet to be clarified. Recently, Zhou *et al.*^39^ reported that the HDAC inhibitor belinostat showed synergistic anti-AML efficacy with pevonedistat by disrupting the DNA damage response. Since LSD1 and HDAC interact with each other through complex formation with CoREST in hematopoietic cells,^5^ it would be of interest to investigate whether similar modes of action operate with LSD1 and HDAC inhibitors in combination with pevonedistat.

In this article, we report that a synergistic interaction between the LSD1 inhibitor T-3775440 and the NAE inhibitor pevonedistat yielded significant anti-AML effects including complete remission in preclinical erythroid leukemia models. Erythroid leukemia is rare (2–4% of AML) but highly refractory to conventional chemotherapy; there is therefore a considerable unmet medical need for effective treatments. Our data, including the antileukemic effects on erythroid leukemia containing cytarabine-resistant TF-1a cells, may be considered promising. Mechanistically, cotreatment with these two agents induced cell transdifferentiation cooperatively, thereby inhibiting cell proliferation. Moreover, pevonedistat-mediated rereplication contributed functionally to the combination with T-3775440 to promote cell death. Two other LSD1 inhibitors, ORY-1001 and GSK2879552, have undergone clinical trials for the treatment of patients with AML (EudraCT number: 2013-002447-29; ClinicalTrials.gov identifier: NCT02177812). Since pevonedistat has been reported to show modest clinical activity in a subset of AML patients,^19^ our findings indicate that the LSD1/NAE inhibitor combination strategy is worth consideration for the treatment of AML.

## Materials and methods

### Cell culture and reagents

The human AML cell line TF-1a was purchased from the American Type Culture Collection (ATCC, Manassas, VA, USA; CRL-2451) in 2008. TF-1a cells and their derivatives were cultivated in RPMI1640 medium containing 10% fetal bovine serum, and maintained in an incubator at 37 °C and 5% CO_2_. Culture methods for other cell lines are available in Supplementary Table 5. TF-1a and MOLM-16 were authenticated by short tandem repeat DNA profiling in 2016. Mycoplasma test was performed by Central Institute for Experimental Animals (Kawasaki, Japan) and all cell lines were confirmed to be negative for mycoplasma. A cytarabine-resistant TF-1a (TF-1a/Ara-C) cell line was developed from parental TF-1a cells by stepwise exposure to increasing concentrations of cytarabine. The resulting TF-1a/Ara-C cells were highly resistant to cytarabine (half-maximal inhibitory concentration value >10 μm) compared with the parental TF-1a cells (half-maximal inhibitory concentration value=0.053 μm). The LSD1 inhibitor T-3775440 and the NAE inhibitor pevonedistat were synthesized by Takeda Pharmaceutical Company (Fujisawa, Japan; Cambridge, MA, USA).

### Establishment of TF-1a-luc stable cell line

TF-1-a cells were seeded into 6-well plates and left overnight to attach in media containing 0.1% fetal bovine serum. pGL-CMV-luc plasmids were transfected using FuGene HD transfection reagent (Promega, Madison, WI, USA) according to the manufacturer’s instructions, at reagent-to-DNA ratios of 5:2. After transfection, cells were cultured in growth medium without antibiotics for 2 days and then in medium containing 350 μg/ml G418 (Life Technologies, Waltham, MA, USA) for selection. Stable clone cell mixtures (1 × 10^2^), obtained following 3 weeks of selection, were reseeded in 6-well dishes with methylcellulose-based semisolid medium (ClonaCell-TCS medium; Stemcell Technologies, Vancouver, BC, Canada) containing 350 μg/ml G418 to select for transformed clones. After 1–2 weeks, individual colonies were picked and grown in 96-well plates with TCS medium containing G418. The clone with the highest luciferase activity was selected and expanded for further experiments.

### Cell proliferation assay

Cells were plated in tissue culture plates and test compounds were added simultaneously. After the treatment period, cells were lysed with CellTiter Glo (Promega) and the luminescent signal was measured using an ARVO MX1420 Microplate Reader (Perkin-Elmer, Waltham, MA, USA).

### Analysis of drug combination effects

Calculation of combination metrics was performed as described previously.^40^ Briefly, a nine- parameter response surface model was fitted to the relationship between normalized viability and drug concentration, after which an isobologram analysis was used to determine the effects of drug combinations.^41^ To quantify the combined effects of the two drugs, the CI with the concentrations of the single agents and combination that gave a normalized viability of 50% was computed.^42, 43^ A CI value below 0.7 was classified as synergy, whereas a value above 1.3 was classified as subadditivity. A value in the range 0.7–1.3 was considered as additivity. Where the maximum inhibition by a single agent was <50%, nonlinear blending^44^ were computed to determine the synergy. A blending value above 20 was classified as synergy, whereas a value above −20 was classified as antagonism.

### Western blotting

Whole-cell extracts or immunoprecipitates were treated with 1 × Laemmli sample buffer (Tris-HCl 125 mm, pH 7.5, 1% sodium dodecyl sulfate, 20% glycerol) and fractionated by sodium dodecyl sulfate–polyacrylamide gel electrophoresis. The proteins were then transferred to nitrocellulose membranes using an iBlot Transfer Stack and iBlot Gel Transfer Device (Thermo Fisher Scientific, Waltham, MA, USA). After incubation with StartingBlock T20 (phosphate-buffered saline) blocking buffer (Pierce Biotechnology, Waltham, MA, USA), membranes were labeled with primary antibodies overnight, followed by incubation with horseradish peroxidase-conjugated secondary antibodies (Cell Signaling Technology, Danvers, MA, USA). Membranes were incubated with ImmunoStar Zeta (Wako, Osaka, Japan) and signals were detected using ImageQuant LAS-3000 (Fujifilm, Tokyo, Japan).

The following antibodies were used for western blotting analysis: CDT1 (sc-365305; Santa Cruz Biotechnology, Dallas, TX, USA), cleaved PARP (9541; Cell Signaling Technology), γH2AX (2577; Cell Signaling Technology), GAPDH (2118; Cell Signaling Technology), GATA1 (3535; Cell Signaling Technology), PU.1 (2258; Cell Signaling Technology) and c-Jun (9165; Cell Signaling Technology).

### Cell cycle analysis

For measurement of DNA content to assess cell cycle distribution, cells were incubated with 70% ethanol/phosphate-buffered saline (v/v) overnight. Fixed cells were stained with propidium iodide and analyzed using a FACSCalibur or FACSVerse System (Becton-Dickinson, Franklin Lakes, NJ, USA).

### Quantitative reverse transcription–polymerase chain reaction analysis and microarray

Following the designated treatment, total RNA was isolated from cells and purified using an RNeasy Mini Kit (Qiagen, Hilden, Germany). Reverse transcription (RT) reactions were performed using a Verso cDNA Synthesis Kit (Thermo Fisher Scientific). Quantitative real-time PCR analysis was performed with a ViiA7 System (Applied Biosystems, Foster City, CA, USA) and TaqMan Fast Advanced Master Mix with TaqMan probes against indicated genes (Applied Biosystems). The 2^–ΔΔCt^ method was applied to analyze the data, using *GAPDH* mRNA expression as an internal control. The normalized abundance of target mRNAs was expressed relative to the corresponding value for cells treated with dimethyl sulfoxide (DMSO) or negative control siRNAs. The following TaqMan probes were used for quantitative RT–PCR (RT–PCR) analysis: *LSD1* (KDM1A, Hs01002741_m1), *GFI1B* (Hs01062469_m1), *GFI1* (Hs01115757_m1), *DTL* (DTL, Hs00978565_m1) and *GAPDH* (Hs02758991_g1).

For microarray analysis, total RNA was purified as described above and the quality of RNA was verified using an Agilent 2100 Bioanalyzer (Agilent Technologies, Santa Clara, CA, USA). RNA was labeled and hybridized to Agilent SurePrint G3 Human Gene Expression 8 × 60 K arrays by Macrogen Company (Seoul, South Korea). Microarray data have been deposited in NCBI GEO (accession number: GSE89637). To examine transcriptome data at the level of gene signatures, gene set enrichment analysis was applied to the microarray data.^45^ The reference signatures used in the analysis were generated from data published elsewhere.^46^

### siRNA transfection

The following siRNAs targeting each gene were obtained: siCTRL (D-001810-10; Dharmacon, Lafayette, CO, USA), LSD1 no. 1 (L-009223-00; Dharmacon), LSD1 no. 2 (118783; Ambion, Waltham, MA, USA), GFI1B no. 1 (s15850; Ambion), GFI1B no. 2 (s15851; Ambion), GFI1 no. 1 (s5706; Ambion), GFI1 no. 2 (s5707; Ambion), and DTL (s28248; Ambion). siRNAs were transfected into cells using GenomeONE-Si (Ishihara Sangyo, Osaka, Japan), or formulated into lipid-based nanoparticles.

### Subcutaneous tumor xenograft models

All animal experiments were conducted in compliance with the guidelines of the Takeda Institutional Animal Care and Use Committee (IACUC; approval number, AU-00006241) in a facility accredited by the American Association for Accreditation of Laboratory Animal Care (AAALAC). Female C.B17/Icr-scid/scid Jcl mice (CLEA Japan, Tokyo, Japan) were maintained under specific pathogen-free conditions. AML cells were subcutaneously inoculated with Matrigel into the left flank of 6- to 7-week-old mice (day 0). Mice were randomized when the mean tumor volume reached ~120–180 mm^3^. Mice were then treated with vehicle, T-3775440 (*per os*), pevonedistat (subcutaneous), cytarabine (intraperitoneal), azacitidine (subcutaneous) or combination treatment. Tumor volume was measured twice weekly using Vernier calipers and calculated as (length × width^2^) × 0.5. The percentage treated/control ratio (T/C %) was calculated by dividing the change in tumor volume in the treated mice by the change in volume in mice administered vehicle. Statistical comparisons were carried out using the one-tailed Williams’ test or Aspin–Welch’s *t*-test (*P*<0.025 or *P*<0.05 were considered statistically significant, respectively).

### AML cell dissemination model

As a dissemination model, TF-1a-luc cells were inoculated via the tail vein into 7-week-old female NOG (NOD.Cg-Prkdcscid Il2rgtm1Sug/Jic) mice (1 × 10^6^ cells per mouse, day 0). The mice (CLEA) were maintained under specific pathogen-free conditions and used in compliance with the guidelines of the Takeda IACUC (approval number, AU-00010345). Administration of T-3775440 alone (*per os*), pevonedistat alone (subcutaneous), T-3775440/pevonedistat in combination or vehicle was initiated 10 days after cell inoculation (day 10). Leukemic cell growth was monitored based on emitted bioluminescence (photons/s) 10 min after intraperitoneal administration of d-luciferin (150 mg/kg) using the *In Vivo* Imaging System (Xenogen, Waltham, MA, USA). Mice reaching the humane end points were killed. Statistical analysis was performed by a log-rank test using prism (GraphPad Prism Software, La Jolla, CA, USA).

### Statistical analysis

The *in vitro* experiments were performed in duplicate or triplicate. Statistical significance was determined using multiple comparison procedures, such as Dunnett's multiple comparison test, as described in the figure legends. A log-rank test was performed to compare survival curves (*P*<0.00555 after the Bonferroni correction was considered statistically significant). GraphPad Prism Software (Version 5; GraphPad Software Inc., La Jolla, CA, USA) was used for the analyses.

## Publisher’s note

Springer Nature remains neutral with regard to jurisdictional claims in published maps and institutional affiliations.

## Figures and Tables

**Table 1:** Synergy score of T-3775440/pevonedistat combination in an AML cell panel

*Cell line*	*Meaning*	*Blending synergy*	*Combination index*	*Incubation (h)*	*FAB*
TF-1a	Synergy	36.5	0.3	72	M6
TF-1a/Ara-C	Synergy	34.9	NA	72	M6
NB4	Synergy	33.5	NA	120	M3
Kasumi-1	Synergy	31.8	0.45	120	M2
MOLM-16	Synergy	28.4	NA	120	M7
HL-60/MX2	Synergy	23.3	0.42	120	M2
HL-60	Synergy	19.6	0.41	120	M2
HEL92.1.7	Additivity	14.6	NA	72	M6
OCI-M2	Additivity	10.9	0.81	72	M1
GF-D8	Additivity	8.6	NA	168	M7
CMK-11-5	Additivity	4.3	0.96	72	M5
THP-1	Additivity	−5.5	NA	144	M4
OCI-AML3	Additivity	−7.9	NA	168	M4
EOL-1	Additivity	−17.3	NA	120	Eosinophilic
CMK-86	Subadditivity	−27.6	1.73	72	M7

Abbreviations: AML, acute myeloid leukemia; CI, combination index; FAB, French–American–British Classification; NA, not applicable. CI values in the range 0–0.7 and 0.7–1.3 are classified as synergy and additivity, respectively. When CI values were not associated, nonlinear blending values >20 and between −20 and +20 were classified as synergy and additivity, respectively. The experiments were conducted in duplicate for TF-1a, TF-1a/Ara-C, NB4, Kasumi-1, HL-60/MX2, HL-60, OCI-M2, GF-D8, OCI-AML3 and in triplicate for MOLM-16, HEL92.1.7, CMK-11–5, THP-1, EOL-1, CMK-86.

## References

[bib1] American Cancer Society Cancer Facts & Figures 2016, https://www.cancer.org/research/cancer-facts-statistics/all-cancer-facts-figures/cancer-facts-figures-2016.html.

[bib2] Shi Y, Lan F, Matson C, Mulligan P, Whetstine JR, Cole PA et al. Histone demethylation mediated by the nuclear amine oxidase homolog LSD1. Cell 2004; 119: 941–953. 15620353 10.1016/j.cell.2004.12.012

[bib3] Metzger E, Wissmann M, Yin N, Muller JM, Schneider R, Peters AH et al. LSD1 demethylates repressive histone marks to promote androgen-receptor-dependent transcription. Nature 2005; 437: 436–439. 16079795 10.1038/nature04020

[bib4] Lee MG, Wynder C, Cooch N, Shiekhattar R. An essential role for CoREST in nucleosomal histone 3 lysine 4 demethylation. Nature 2005; 437: 432–435. 16079794 10.1038/nature04021

[bib5] Saleque S, Kim J, Rooke HM, Orkin SH. Epigenetic regulation of hematopoietic differentiation by Gfi-1 and Gfi-1b is mediated by the cofactors CoREST and LSD1. Mol Cell 2007; 27: 562–572. 17707228 10.1016/j.molcel.2007.06.039

[bib6] Niebel D, Kirfel J, Janzen V, Holler T, Majores M, Gutgemann I. Lysine-specific demethylase 1 (LSD1) in hematopoietic and lymphoid neoplasms. Blood 2014; 124: 151–152. 24993879 10.1182/blood-2014-04-569525

[bib7] Lynch JT, Harris WJ, Somervaille TC. LSD1 inhibition: a therapeutic strategy in cancer? Expert Opin Ther Targets 2012; 16: 1239–1249. 22957941 10.1517/14728222.2012.722206

[bib8] Harris WJ, Huang X, Lynch JT, Spencer GJ, Hitchin JR, Li Y et al. The histone demethylase KDM1A sustains the oncogenic potential of MLL-AF9 leukemia stem cells. Cancer Cell 2012; 21: 473–487. 22464800 10.1016/j.ccr.2012.03.014

[bib9] Mohammad HP, Smitheman KN, Kamat CD, Soong D, Federowicz KE, Van Aller GS et al. A DNA hypomethylation signature predicts antitumor activity of LSD1 inhibitors in SCLC. Cancer Cell 2015; 28: 57–69. 26175415 10.1016/j.ccell.2015.06.002

[bib10] McGrath JP, Williamson KE, Balasubramanian S, Odate S, Arora S, Hatton C et al. Pharmacological inhibition of the histone lysine demethylase KDM1A suppresses the growth of multiple acute myeloid leukemia subtypes. Cancer Res 2016; 76: 1975–1988. 26837761 10.1158/0008-5472.CAN-15-2333

[bib11] Ishikawa Y, Gamo K, Yabuki M, Takagi S, Toyoshima K, Nakayama K et al. A novel LSD1 inhibitor T-3775440 disrupts GFI1B-containing complex leading to transdifferentiation and impaired growth of AML cells. Mol Cancer Ther 2017; 16: 273–284. 27903753 10.1158/1535-7163.MCT-16-0471

[bib12] Schenk T, Chen WC, Gollner S, Howell L, Jin L, Hebestreit K et al. Inhibition of the LSD1 (KDM1A) demethylase reactivates the all-*trans*-retinoic acid differentiation pathway in acute myeloid leukemia. Nat Med 2012; 18: 605–611. 22406747 10.1038/nm.2661 PMC3539284

[bib13] Fiskus W, Sharma S, Shah B, Portier BP, Devaraj SG, Liu K et al. Highly effective combination of LSD1 (KDM1A) antagonist and pan-histone deacetylase inhibitor against human AML cells. Leukemia 2014; 28: 2155–2164. 24699304 10.1038/leu.2014.119 PMC4739780

[bib14] Soucy TA, Smith PG, Milhollen MA, Berger AJ, Gavin JM, Adhikari S et al. An inhibitor of NEDD8-activating enzyme as a new approach to treat cancer. Nature 2009; 458: 732–776. 19360080 10.1038/nature07884

[bib15] Nawrocki ST, Griffin P, Kelly KR, Carew JS. MLN4924: a novel first-in-class inhibitor of NEDD8-activating enzyme for cancer therapy. Expert Opin Investig Drugs 2012; 21: 1563–1573. 10.1517/13543784.2012.707192 22799561

[bib16] Nakayama KI, Nakayama K. Ubiquitin ligases: cell-cycle control and cancer. Nat Rev Cancer 2006; 6: 369–381. 16633365 10.1038/nrc1881

[bib17] Skaar JR, Pagan JK, Pagano M. SCF ubiquitin ligase-targeted therapies. Nat Rev Drug Discov 2014; 13: 889–903. 25394868 10.1038/nrd4432 PMC4410837

[bib18] Swords RT, Kelly KR, Smith PG, Garnsey JJ, Mahalingam D, Medina E et al. Inhibition of NEDD8-activating enzyme: a novel approach for the treatment of acute myeloid leukemia. Blood 2010; 115: 3796–3800. 20203261 10.1182/blood-2009-11-254862

[bib19] Swords RT, Erba HP, DeAngelo DJ, Bixby DL, Altman JK, Maris M et al. Pevonedistat (MLN4924), a first-in-class NEDD8-activating enzyme inhibitor, in patients with acute myeloid leukaemia and myelodysplastic syndromes: a phase 1 study. Br J Haematol 2015; 169: 534–543. 25733005 10.1111/bjh.13323

[bib20] Kee Y, Huang M, Chang S, Moreau LA, Park E, Smith PG et al. Inhibition of the Nedd8 system sensitizes cells to DNA interstrand cross-linking agents. Mol Cancer Res 2012; 10: 369–377. 22219386 10.1158/1541-7786.MCR-11-0497 PMC3307881

[bib21] Blank JL, Liu XJ, Cosmopoulos K, Bouck DC, Garcia K, Bernard H et al. Novel DNA damage checkpoints mediating cell death induced by the NEDD8-activating enzyme inhibitor MLN4924. Cancer Res 2013; 73: 225–234. 23100467 10.1158/0008-5472.CAN-12-1729

[bib22] Milhollen MA, Narayanan U, Soucy TA, Veiby PO, Smith PG, Amidon B. Inhibition of NEDD8-activating enzyme induces rereplication and apoptosis in human tumor cells consistent with deregulating CDT1 turnover. Cancer Res 2011; 71: 3042–3051. 21487042 10.1158/0008-5472.CAN-10-2122

[bib23] Khalife J, Radomska HS, Santhanam R, Huang X, Neviani P, Saultz J et al. Pharmacological targeting of miR-155 via the NEDD8-activating enzyme inhibitor MLN4924 (Pevonedistat) in FLT3-ITD acute myeloid leukemia. Leukemia 2015; 29: 1981–1992. 25971362 10.1038/leu.2015.106 PMC4868182

[bib24] Saleque S, Cameron S, Orkin SH. The zinc-finger proto-oncogene Gfi-1b is essential for development of the erythroid and megakaryocytic lineages. Genes Dev 2002; 16: 301–306. 11825872 10.1101/gad.959102 PMC155332

[bib25] Vassen L, Fiolka K, Mahlmann S, Moroy T. Direct transcriptional repression of the genes encoding the zinc-finger proteins Gfi1b and Gfi1 by Gfi1b. Nucleic Acids Res 2005; 33: 987–998. 15718298 10.1093/nar/gki243 PMC549408

[bib26] Lin JJ, Milhollen MA, Smith PG, Narayanan U, Dutta A. NEDD8-targeting drug MLN4924 elicits DNA rereplication by stabilizing Cdt1 in S phase, triggering checkpoint activation, apoptosis, and senescence in cancer cells. Cancer Res 2010; 70: 10310–10320. 21159650 10.1158/0008-5472.CAN-10-2062 PMC3059213

[bib27] Tenen DG. Disruption of differentiation in human cancer: AML shows the way. Nat Rev Cancer 2003; 3: 89–101. 12563308 10.1038/nrc989

[bib28] Liao H, Liu XJ, Blank JL, Bouck DC, Bernard H, Garcia K et al. Quantitative proteomic analysis of cellular protein modulation upon inhibition of the NEDD8-activating enzyme by MLN4924. Mol Cell Proteomics 2011; 10: M111.009183. 10.1074/mcp.M111.009183 PMC3226404 21873567

[bib29] Nateri AS, Riera-Sans L, Da Costa C, Behrens A. The ubiquitin ligase SCFFbw7 antagonizes apoptotic JNK signaling. Science (New York, NY) 2004; 303: 1374–1378. 10.1126/science.1092880 14739463

[bib30] Sarantopoulos J, Shapiro GI, Cohen RB, Clark JW, Kauh JS, Weiss GJ et al. Phase I study of the investigational NEDD8-activating enzyme inhibitor pevonedistat (TAK-924/MLN4924) in patients with advanced solid tumors. Clin Cancer Res 2016; 22: 847–857. 26423795 10.1158/1078-0432.CCR-15-1338

[bib31] Shimizu R, Engel JD, Yamamoto M. GATA1-related leukaemias. Nat Rev Cancer 2008; 8: 279–287. 18354416 10.1038/nrc2348

[bib32] Zhang P, Behre G, Pan J, Iwama A, Wara-Aswapati N, Radomska HS et al. Negative cross-talk between hematopoietic regulators: GATA proteins repress PU.1. Proc Natl Acad Sci USA 1999; 96: 8705–8710. 10411939 10.1073/pnas.96.15.8705 PMC17580

[bib33] Rekhtman N, Radparvar F, Evans T, Skoultchi AI. Direct interaction of hematopoietic transcription factors PU.1 and GATA-1: functional antagonism in erythroid cells. Genes Dev 1999; 13: 1398–1411. 10364157 10.1101/gad.13.11.1398 PMC316770

[bib34] Burda P, Laslo P, Stopka T. The role of PU.1 and GATA-1 transcription factors during normal and leukemogenic hematopoiesis. Leukemia 2010; 24: 1249–1257. 20520638 10.1038/leu.2010.104

[bib35] Tan M, Li Y, Yang R, Xi N, Sun Y. Inactivation of SAG E3 ubiquitin ligase blocks embryonic stem cell differentiation and sensitizes leukemia cells to retinoid acid. PLoS ONE 2011; 6: e27726. 22110742 10.1371/journal.pone.0027726 PMC3217012

[bib36] Behre G, Whitmarsh AJ, Coghlan MP, Hoang T, Carpenter CL, Zhang DE et al. c-Jun is a JNK-independent coactivator of the PU.1 transcription factor. J Biol Chem 1999; 274: 4939–4946. 9988737 10.1074/jbc.274.8.4939

[bib37] Burda P, Curik N, Kokavec J, Basova P, Mikulenkova D, Skoultchi AI et al. PU.1 activation relieves GATA-1-mediated repression of Cebpa and Cbfb during leukemia differentiation. Mol Cancer Res 2009; 7: 1693–1703. 19825991 10.1158/1541-7786.MCR-09-0031 PMC3193075

[bib38] Mosammaparast N, Kim H, Laurent B, Zhao Y, Lim HJ, Majid MC et al. The histone demethylase LSD1/KDM1A promotes the DNA damage response. J Cell Biol 2013; 203: 457–470. 24217620 10.1083/jcb.201302092 PMC3824007

[bib39] Zhou L, Chen S, Zhang Y, Kmieciak M, Leng Y, Li L et al. The NAE inhibitor pevonedistat interacts with the HDAC inhibitor belinostat to target AML cells by disrupting the DDR. Blood 2016; 127: 2219–2230. 26851293 10.1182/blood-2015-06-653717 PMC4859196

[bib40] Garcia K, Blank JL, Bouck DC, Liu XJ, Sappal DS, Hather G et al. Nedd8-activating enzyme inhibitor MLN4924 provides synergy with mitomycin C through interactions with ATR, BRCA1/BRCA2, and chromatin dynamics pathways. Mol Cancer Ther 2014; 13: 1625–1635. 24672057 10.1158/1535-7163.MCT-13-0634

[bib41] Minto CF, Schnider TW, Short TG, Gregg KM, Gentilini A, Shafer SL. Response surface model for anesthetic drug interactions. Anesthesiology 2000; 92: 1603–1616. 10839909 10.1097/00000542-200006000-00017

[bib42] Chou TC, Talalay P. Quantitative analysis of dose-effect relationships: the combined effects of multiple drugs or enzyme inhibitors. Adv Enzyme Regul 1984; 22: 27–55. 6382953 10.1016/0065-2571(84)90007-4

[bib43] Berenbaum MC. The expected effect of a combination of agents: the general solution. J Theor Biol 1985; 114: 413–431. 4021503 10.1016/s0022-5193(85)80176-4

[bib44] Peterson JJ, Novick SJ. Nonlinear blending: a useful general concept for the assessment of combination drug synergy. J Recept Signal Transduct Res 2007; 27: 125–146. 17613725 10.1080/10799890701417576

[bib45] Subramanian A, Tamayo P, Mootha VK, Mukherjee S, Ebert BL, Gillette MA et al. Gene set enrichment analysis: a knowledge-based approach for interpreting genome-wide expression profiles. Proc Natl Acad Sci USA 2005; 102: 15545–15550. 16199517 10.1073/pnas.0506580102 PMC1239896

[bib46] Novershtern N, Subramanian A, Lawton LN, Mak RH, Haining WN, McConkey ME et al. Densely interconnected transcriptional circuits control cell states in human hematopoiesis. Cell 2011; 144: 296–309. 21241896 10.1016/j.cell.2011.01.004 PMC3049864

