# Correction: Synergistic anti-AML effects of the LSD1 inhibitor T-3775440 and the NEDD8-activating enzyme inhibitor pevonedistat via transdifferentiation and DNA rereplication

**DOI:** 10.1038/s41389-019-0160-0

**Published:** 2019-09-11

**Authors:** Y. Ishikawa, K. Nakayama, M. Morimoto, A. Mizutani, A. Nakayama, K. Toyoshima, A. Hayashi, S. Takagi, R. Dairiki, H. Miyashita, S. Matsumoto, K. Gamo, T. Nomura, K. Nakamura

**Affiliations:** 1 Oncology Drug Discovery Unit, Pharmaceutical Research Division, Takeda Pharmaceutical Company Limited, Fujisawa, Japan; 2 Integrated Technology Research Laboratories, Pharmaceutical Research Division, Takeda Pharmaceutical Company Limited, Fujisawa, Japan

**Keywords:** Breast cancer, Targeted therapies

**Correction to: Oncogenesis**

10.1038/oncsis.2017.76 Published online 11 September 2017

The FAB classification of OCI-M2, GF-D8, CMK-11-5, and THP-1 cell lines in Table 1 was wrong. This error has been fixed in the revised table.

**Table 1:**
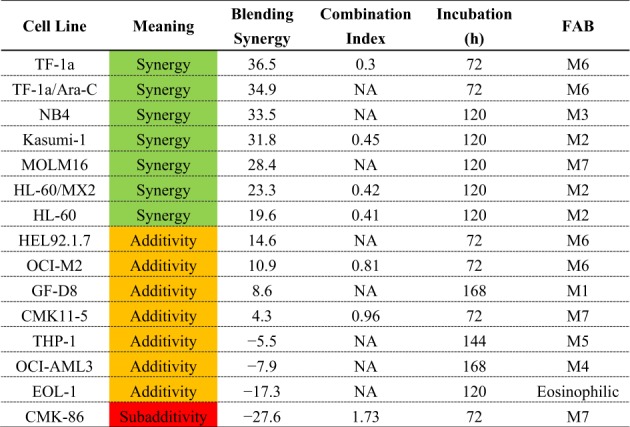
Synergy score of T-3775440/pevonedistat combination in an AML cell panel

NOTE: Combination index (CI) values in the range 0–0.7 and 0.7–1.3 are classified as synergy and additivity, respectively. When CI values were not associated, nonlinear blending values greater than 20 and between −20 and +20 were classified as synergy and additivity, respectively. Heat maps are color-coded based on the combination effects: Green, synergy; orange, additive; red, subadditive.

