# Supplementary material for: Synergistic anti-AML effects of the LSD1 inhibitor T-3775440 and the NEDD8-activating enzyme inhibitor pevonedistat via transdifferentiation and DNA rereplication
Source: Oncogenesis. 2017 Sep 11;6(9):e377–. doi: 10.1038/oncsis.2017.76 (PMC5623902; doi:10.1038/oncsis.2017.76)
Supplement: Supplementary Tables [file oncsis201776x1.docx]

Supplementary Table 1. Effect of T-3775440 on the sensitivity of TF-1a cells to pevonedistat (related to Fig. 1a).

| T-3775440 | Relative cell proliferation (%) | | EC_50_ of pevonedistat (nmol/L) | 95% Confidence intervals (nmol/L) |
| --- | --- | --- | --- | --- |
|  | Bottom | Top |  |  |
| 0 nM | -3.0 | 96.2 | 770 | 583–1015 |
| 4.6 nM | 1.9 | 83.9 | 326^***^ | 283–375 |
| 21.5 nM | 3.4 | 59.5 | 215^***^ | 191–241 |
| 100 nM | 3.1 | 53.9 | 167^***^ | 152–185 |

NOTE: TF-1a cells were co-treated with pevonedistat and T-3775440 at the indicated concentrations for 72 h. EC_50_ values and their 95% confidence intervals were calculated using nonlinear regression analysis of the percentage inhibition. One-way ANOVA along with post-Dunnett's multiple comparison test were performed to compare the EC_50_ values obtained from the nonlinear regression analysis [^***^*P* < 0.001, versus control (0 nM)].

Supplementary Table 2. Effect of GFI1 family protein knockdown on the sensitivity of TF-1a cells to pevonedistat (related to Fig. 1e).

| siRNA | Relative cell proliferation (%) | | EC_50_ of pevonedistat (nmol/L) | 95% Confidence intervals (nmol/L) |
| --- | --- | --- | --- | --- |
|  | Bottom | Top |  |  |
| siControl | 0.3 | 97.1 | 427 | 365–499 |
| siLSD1#1 | 2.2 | 89.7 | 168^*^ | 150–187 |
| siLSD1#2 | 2.4 | 89.1 | 153^*^ | 140–166 |
| siGFI1B#1 | 2.2 | 67.2 | 196^*^ | 170–227 |
| siGFI1B#2 | 2.1 | 63.4 | 232^*^ | 208–259 |
| siGFI1#1 | -0.3 | 90.2 | 395 | 317–492 |
| siGFI1#2 | -0.4 | 88.7 | 402 | 336–480 |

NOTE: TF-1a cells were treated with the indicated siRNAs for 6 h and re-plated. After overnight incubation, cells were treated with pevonedistat for 72 h. For each siRNA treatment, the EC_50_ values of pevonedistat were calculated. All assays were done in triplicate (n = 3). EC_50_ values and their 95% confidence intervals were calculated using nonlinear regression analysis of the percentage inhibition. siLSD1 and siGFI1B, but not siGFI1, augmented the anti-proliferative activity of pevonedistat in TF-1a cells. One-way ANOVA and post-Dunnett's multiple comparison test were performed to compare the EC_50_ values obtained from the nonlinear regression analysis (^*^*P* < 0.05, versus siControl).

Supplementary Table 3. Effects of DTL knockdown on the sensitivity of TF-1a cells to T-3775440 (related to Fig. S8b).

| siRNA | Relative cell proliferation (%) | | EC_50_ of pevonedistat (nmol/L) | 95% Confidence intervals (nmol/L) |
| --- | --- | --- | --- | --- |
|  | Bottom | Top |  |  |
| siCTRL | 50.21 | 94.21 | 11.1 | 9.0–13.7 |
| siDTL | 9.653 | 39.34 | 5.1^***^ | 4.1–6.5 |

NOTE: TF-1a cells were treated with the indicated siRNAs for 6 h and re-plated. After incubation overnight, cells were treated with pevonedistat for 72 h. For each siRNA treatment, the EC_50_ values of T-3775440 were calculated. All assays were done in quadruplicate. EC_50_ values and their 95% confidence intervals were calculated using nonlinear regression analysis of the percentage inhibition. Unpaired t test was performed to compare the EC_50_ values obtained from the nonlinear regression analysis (^***^*P* < 0.001, versus siControl).

Supplementary Table 4. Median survival time after administration of T-3775440, pevonedistat, or the indicated combination in the TF-1a-luc disseminated model (related to Fig. 5).

| Group | Median survival (days) | *P* value |
| --- | --- | --- |
| Control | 30 | NA |
| 2.5 mg/kg T-3775440 | 43 | < 0.0001 |
| 5 mg/kg T-3775440 | 46 | < 0.0001 |
| 10 mg/kg T-3775440 | 50 | < 0.0001 |
| 20 mg/kg T-3775440 | 54 | 0.0004 |
| 60 mg/kg pevonedistat | 36 | < 0.0001 |
| T-3775440 (2.5) + pevonedistat (60) | 62 | 0.0004 |
| T-3775440 (5) + pevonedistat (60) | 68 | 0.0004 |
| T-3775440 (10) + pevonedistat (60) | 60 | 0.0004 |
| T-3775440 (20) + pevonedistat (60) | 77 | 0.0004 |

NOTE: Statistical analysis was performed with a Log-rank test (*p* <0.00555 after the Bonferroni correction was considered statistically significant).

Supplementary Table 5. Culture medium information for cell lines used in cell proliferation assays.

| Cell line | Medium | FBS | Supplement |
| --- | --- | --- | --- |
| CMK-11-5 (JCRB) | RPMI 1640 (Wako, 189-02145) | 10% | no |
| CMK-86 (JCRB) | RPMI 1640 (Wako, 189-02145) | 10% | no |
| EOL-1 (DSMZ) | RPMI 1640 (Wako, 189-02145) | 10% | no |
| GF-D8 (DSMZ) | RPMI 1640 (Wako, 189-02145) | 20% | 50 ng/ml rhGM-CSF (Wako, 075-04114) |
| HEL92.1.7(ATCC) | RPMI 1640 (Wako, 189-02145) | 10% | no |
| HL-60 (ATCC) | RPMI 1640 (Wako, 189-02145) | 10% | no |
| HL-60/MX2 (ATCC) | RPMI 1640 (Wako, 189-02145) | 10% | no |
| Kasumi-1 (JCRB) | RPMI 1640 (Wako, 189-02145) | 10% | no |
| MOLM-16 (DSMZ) | RPMI 1640 (Wako, 189-02145) | 20% | no |
| NB4 (DSMZ) | RPMI 1640 (Wako, 189-02145) | 10% | no |
| OCI-AML3 (DSMZ) | MEM α (Wako, 135-15175) | 20% | no |
| OCI-M2 (DSMZ) | IMDM (Gibco, 12440-053) | 20% | no |
| TF-1a (ATCC) | RPMI 1640 (Wako, 189-02145) | 10% | no |
| TF-1a/Ara-C | RPMI 1640 (Wako, 189-02145) | 10% | no |
| THP-1 (ATCC) | RPMI 1640 (Wako, 189-02145) | 10% | no |
| CCRF-CEM (ATCC) | RPMI 1640 (Wako, 189-02145) | 10% | Sodium Pyruvate (Gibco, 11360) |
| MOLT-3 (ATCC) | RPMI 1640 (Wako, 189-02145) | 10% | no |
| RPMI8226 (JCRB) | RPMI 1640 (Wako, 189-02145) | 10% | no |
| KMS28BM (JCRB) | RPMI 1640 (Wako, 189-02145) | 10% | no |
| HepG2 | EMEM (Wako, 051-07615) | 10% | Sodium Pyruvate (Gibco, 11360)  NEAA (Gibco, 11140) |
