# Supplementary material for: Synergistic anti-AML effects of the LSD1 inhibitor T-3775440 and the NEDD8-activating enzyme inhibitor pevonedistat via transdifferentiation and DNA rereplication
Source: Oncogenesis. 2017 Sep 11;6(9):e377–. doi: 10.1038/oncsis.2017.76 (PMC5623902; doi:10.1038/oncsis.2017.76)
Supplement: Supplementary Methods and Figures [file oncsis201776x2.docx]

**Supplementary Information**

**Synergistic Anti-AML Effects of the LSD1 Inhibitor T-3775440 and the NEDD8-Activating Enzyme Inhibitor Pevonedistat via Transdifferentiation and DNA Re-replication**

Y Ishikawa, K Nakayama, M Morimoto, A Mizutani, A Nakayama, K Toyoshima, A Hayashi, S Takagi, R Dairiki, H Miyashita, S Matsumoto, K Gamo, T Nomura, and K Nakamura

One Supplementary Method, 5 Supplementary Tables, and 12 Supplementary Figures

**Supplementary Method**

**AmpliSeq transcriptome analysis**

Deoxycytidine kinase (DCK) mRNA expression was measured using the Ion Ampliseq transcriptome system. DCK expression was normalized to that of GAPDH. Total RNA was extracted from TF-1a and TF-1a/AraC cells using an RNeasy mini kit (Qiagen). A total 10 ng of RNA was reverse transcribed using the Ion AmpliSeq transcriptome human gene expression kit following the manufacturer’s protocol (Thermo Fisher Scientific). The cDNA libraries were amplified and barcoded using an Ion AmpliSeq transcriptome human gene expression core panel and Ion Xpress Barcode Adapter (Thermo Fisher Scientific). The prepared libraries were purified using Agencourt AMPure XP (Beckman Coulter Life Sciences), quantified with the Ion Library TaqMan quantitation kit (Thermo Fisher Scientific), diluted to 75 pM, and pooled equally (9 samples per pool). Emulsion polymerase chain reaction (PCR), enrichment, and loading were performed using an Ion Chef instrument. The templated libraries were then sequenced using an Ion Proton system with an Ion P1 Hi-Q Chef kit and an Ion P1 Chip kit v3 (Thermo Fisher Scientific). The Ion Proton reads were analyzed using the AmpliSeqRNA analysis plugin, v5.2.1.2, in the Torrent Suite Software.

**Supplementary Figures**

**
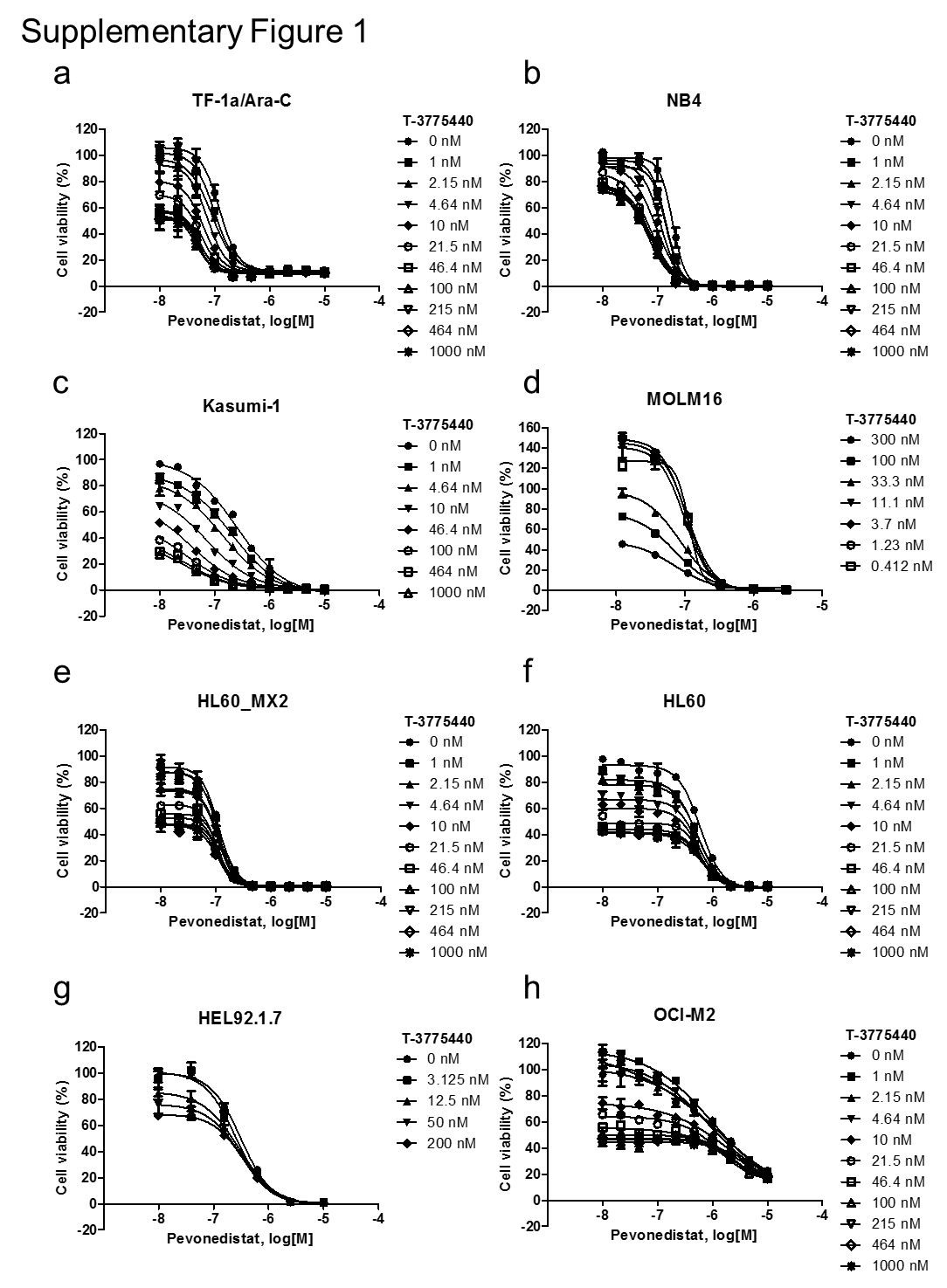
**

Supplementary Figure 1. The combination effects of T-3775440 and pevonedistat in an AML cell panel. (a–n) Cells were treated with pevonedistat in the presence or absence of T-3775440. Viability was measured using CellTiter Glo assay at the time points indicated in Supplementary Table 1. The experiments were performed in duplicate (a-c, e, f, h, i, l) or triplicate (d, g, j, k, m, n). *Values*, means; *bars*, SD.


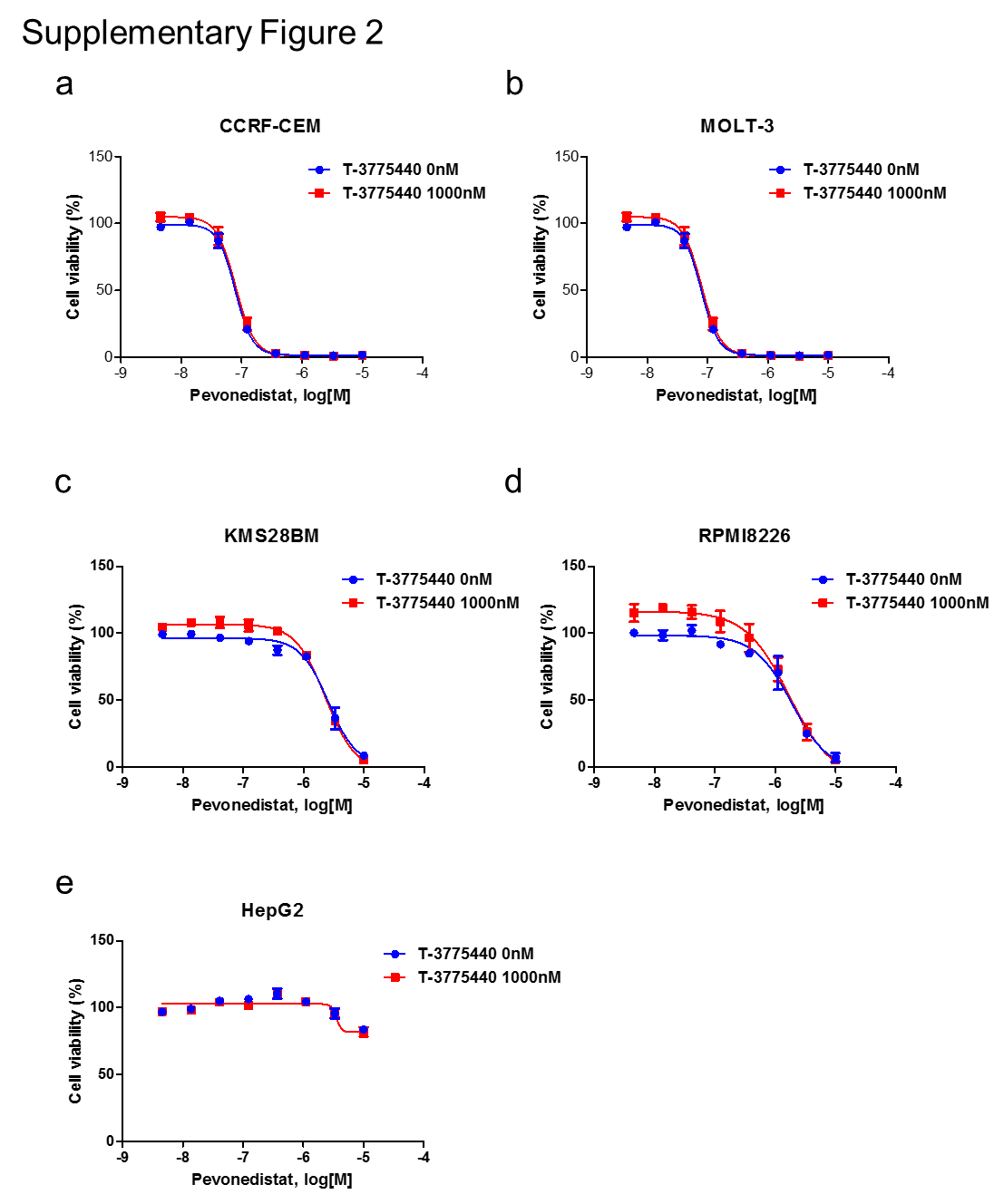


Supplementary Figure 2. Effects of the T-3775440/pevonedistat combination in non-AML cell lines. (a–e) Cells were treated with pevonedistat in the presence or absence of T-3775440. Viability was measured after 120 h of treatment in CCRF-CEM (a), MOLT-3 (b), KMS28BM (c), and RPMI8226 (d) cell lines. (e) For HepG2, viability was determined after 72 h of treatment. Experiments were performed in triplicate. *Values*, means; *bars*, SD.


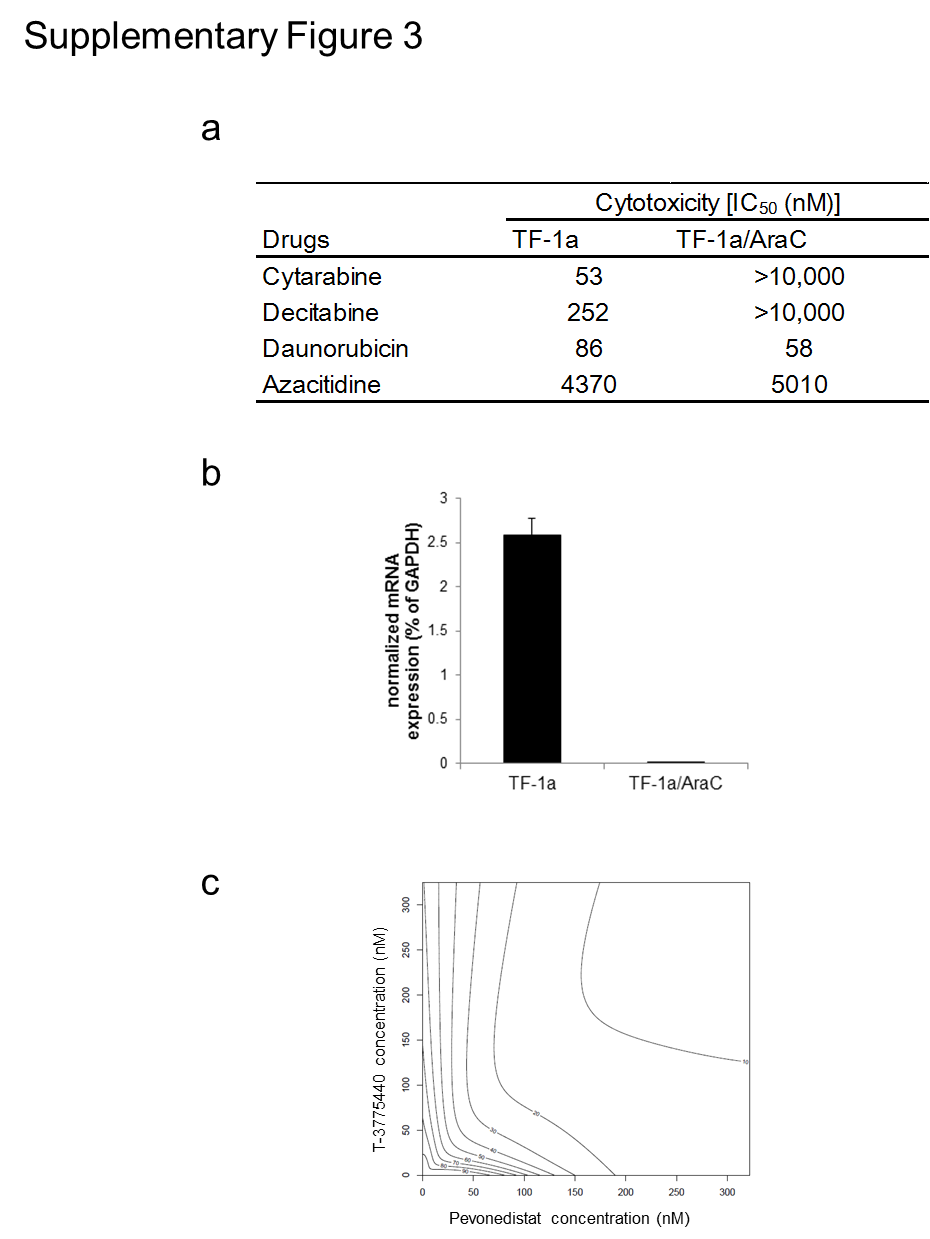


Supplementary Figure 3. The combination effect of T-3775440 and pevonedistat in cytarabine-resistant TF-1a (TF-1a/AraC) cells. The cytarabine-resistant TF-1a (TF-1a/Ara-C) cell line was developed from parental TF-1a cells by “stepwise” exposure to increasing concentrations of cytarabine. (a) TF-1a and TF-1a/AraC cells were treated with each compound and cell viability was measured 72 h later using a CellTiter Glo assay. The IC_50_ values shown (n = 4) were calculated using nonlinear regression analysis. The resultant TF-1a/Ara-C cells were highly resistant to cytarabine (IC_50_ > 10 µM) compared to the parental TF-1a cells (IC_50_ = 0.053 µM). The cross-resistance study revealed that TF-1a/Ara-C cells were as sensitive to daunorubicin and azacitidine as the parental TF-1a cells were. Daunorubicin is a substrate of P-glycoprotein (P-gp), while azacitidine is a substrate of equilibrative nucleoside transporter 1 (hENT1). Therefore, the cytarabine resistance of TF-1a/AraC cells was thought to be independent of either overexpression of P-gP or loss of hENT1 function, which often confers drug resistance by decreasing the drug concentration in AML cells. In contrast, TF-1a/Ara-C cells showed cross-resistance to decitabine. (b) Since both decitabine and cytarabine require deoxycytidine kinase (DCK), a rate-limiting activating enzyme, to exert their anti-leukemic activities, we determined the expression level of DCK. The results revealed that DCK mRNA expression was undetectable in TF-1a/AraC cells. These data suggest that resistance to cytarabine and decitabine is conferred by a deficiency in the activation processes due to DCK downregulation. (c) Isobologram of the T-3775440/pevonedistat combination in TF-1a/AraC cells

Supplementary Figure 4. The combination effects of a panel of anti-leukemic agents in TF-1a cells. (a–j) TF-1a cells were treated with the indicated combination of small molecule inhibitors. Viability was measured after 72 h of treatment using Cell Titer Glo. Experiments were performed in duplicate. *Values*, means; *bars*, SD.

Supplementary Figure 5. The combination of T-3775440 and pevonedistat shows synergistic growth inhibition of Kasumi-1 cells. (a) Kasumi-1 cells were treated with each combination of agents and cell viability was measured after 120 h of treatment. Values represent the combination index (CI) for each combination. Experiments were performed in duplicate. (b) The isobologram of the cotreatment of T-3775440/pevonedistat in Kasumi-1 cells is shown.


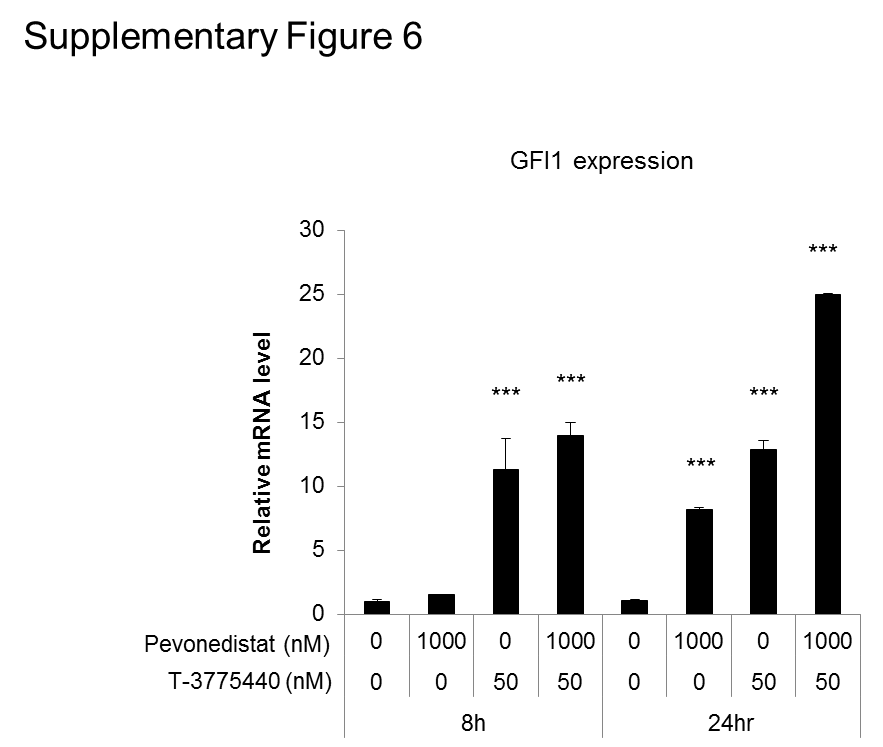


Supplementary Figure 6. Cotreatment with T-3775440/pevonedistat augments GFI1 expression. TF-1a cells were treated with dimethyl sulfoxide (DMSO), 1000 nM pevonedistat, 50 nM T-3775440, or pevonedistat and T-3775440 in combination for 8 or 24 h. Total RNA was purified from the cells and subjected to quantitative reverse transcription – polymerase chain reaction (qRT-PCR) analysis (n=2). Statistical significance was determined using Dunnett's multiple comparison test (^***^*P* < 0.001). *Columns*, means; *bars*, SD.


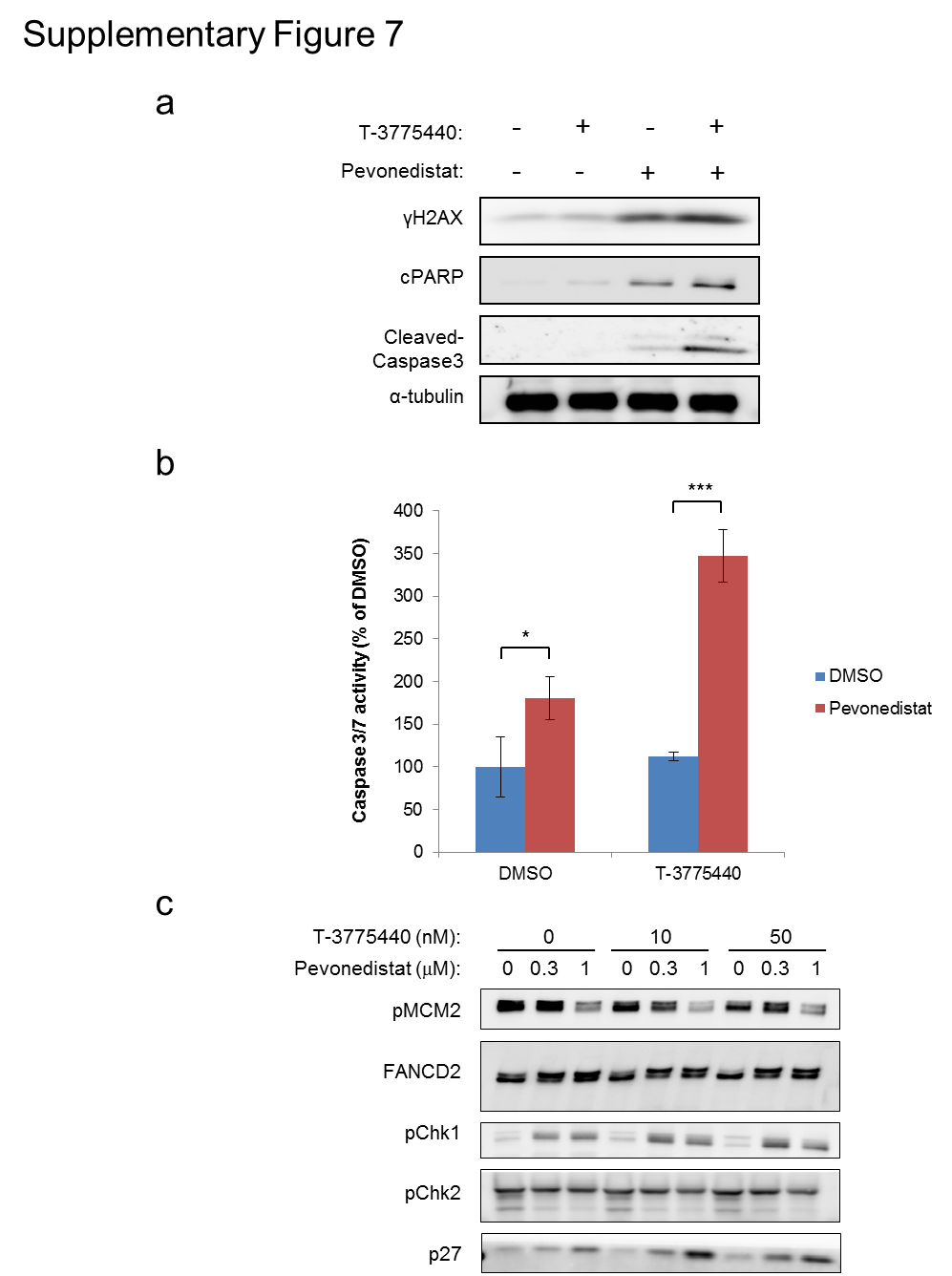


Supplementary Figure 7. Apoptosis induction by co-treatment with T-3775440 and pevonedistat. (a) TF-1a cells were treated with T-3775440 and pevonedistat as indicated for 48 h (T-3775440, 50 nM; Pevonedistat, 1000 nM). Whole cell lysates were prepared and subjected to immunoblotting analysis. Alpha-Tubulin was used as a loading control. (b) TF-1a cells were treated as indicated (T-3775440, 50 nM; Pevonedistat, 100 nM). After 24 h treatment, caspase 3/7 activities were determined using a Caspase-Glo kit (Promega). *Columns*, means; *bars*, SD. n = 3. Bonferroni post hoc test following analysis of variance (ANOVA) was used for the statistical analyses (^*^*P* < 0.05, ^***^*P* < 0.001). (c) TF-1a cells were treated with drugs or dimethyl sulfoxide (DMSO) control for 48 h. Immunoblotting analysis was performed to determine the expression levels of indicated proteins.

Supplementary Figure 8. Knockdown of DTL sensitizes TF-1a cells to T-3775440-induced cell growth inhibition. (a) TF-1a cells were treated with siRNA against DTL or control for 4 h. Forty-eight hours after the initial siRNA treatment, cells were harvested for RNA purification. Changes in *DTL* expression were measured by quantitative reverse transcription-polymerase chain reaction (qRT-PCR). The values represent the means of duplicate samples ± SD. Unpaired t test was employed for statistical analysis (^***^*P* < 0.001). (b) The effects of siRNA targeting DTL on T-3775440-induced growth inhibition in TF-1a cells. Cells were treated with siRNA against DTL or control for 4 h, and after overnight incubation, cells were treated with T-3775440 or dimethyl sulfoxide (DMSO) control for 72 h. Viability was expressed as cell proliferation relative to the siCTRL/DMSO cotreatment control.


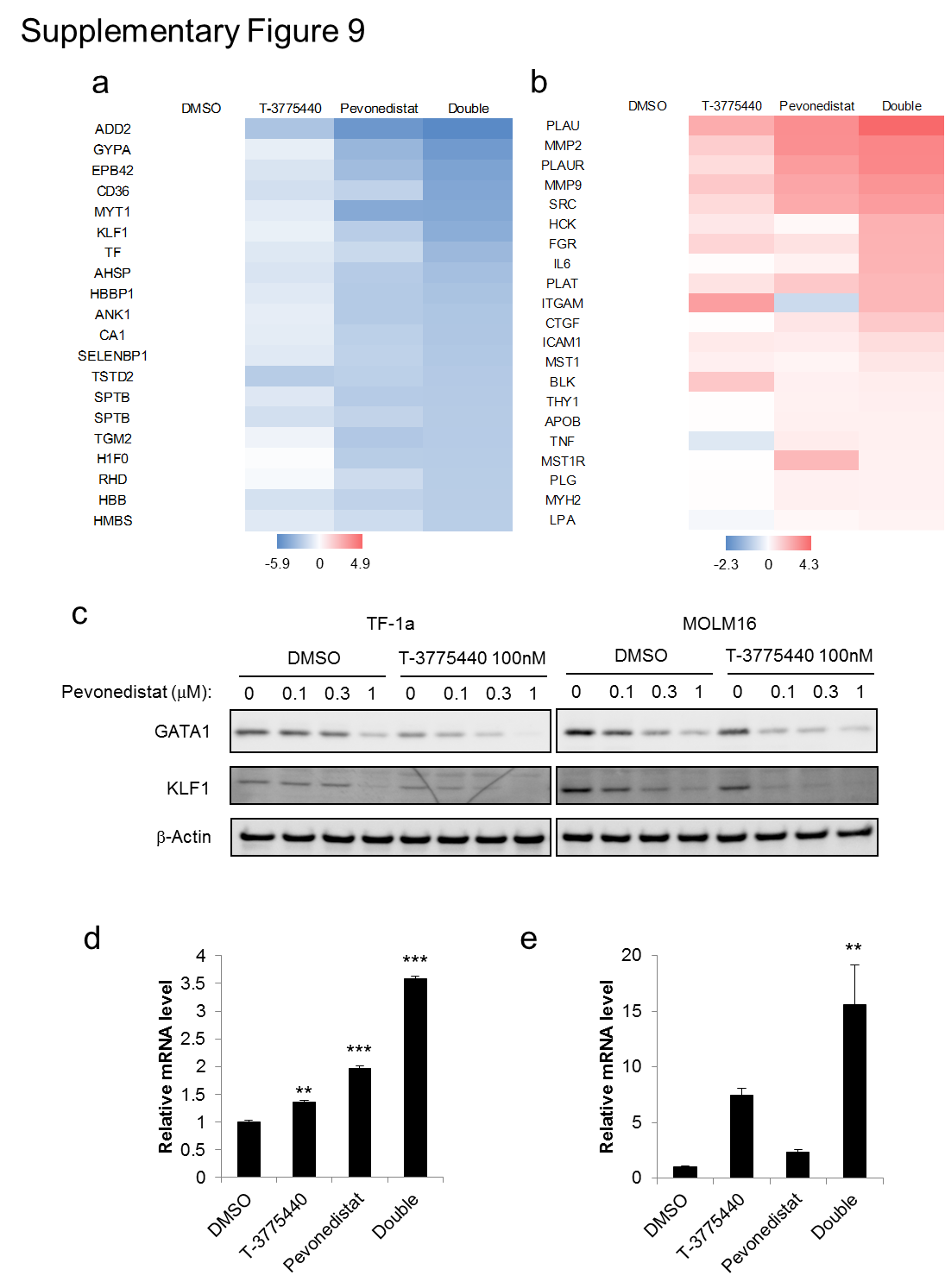


Supplementary Figure 9. Cotreatment with T-3775440 and pevonedistat modulates the expression of differentiation-associated genes. (a, b, d, e) TF-1a cells were treated with vehicle, 50 nM T-3775440, 1000 nM pevonedistat, or T-3775440 and pevonedistat in combination for 24 h. Total RNA was subjected to microarray analysis. A heat map shows fold changes in gene expression of erythroid signature genes (a) and neutrophil signature genes (b). Upregulated and downregulated genes relative to dimethyl sulfoxide (DMSO) control are represented as red and blue, respectively. (c) TF-1a or MOLM16 cells were treated with T-3775440 and pevonedistat as indicated for 24 h. Whole cell lysates were prepared and subjected to immunoblotting analysis. b-Actin was used as a loading control. (d) The mRNA expression level of CEBPA was obtained from the microarray analysis as in (a). (e) Cotreatment with T-3775440/pevonedistat augments each drug-induced CD86 expression. TF-1a cells were treated with DMSO, 300 nM pevonedistat, 10 nM T-3775440, or pevonedistat and T-3775440 in combination for 24 h. Total RNA was purified from the cells and subjected to quantitative reverse transcription – polymerase chain reaction (qRT-PCR) analysis. Bonferroni's multiple comparison test was employed (^**^*P* < 0.01, ^***^*P* < 0.001).

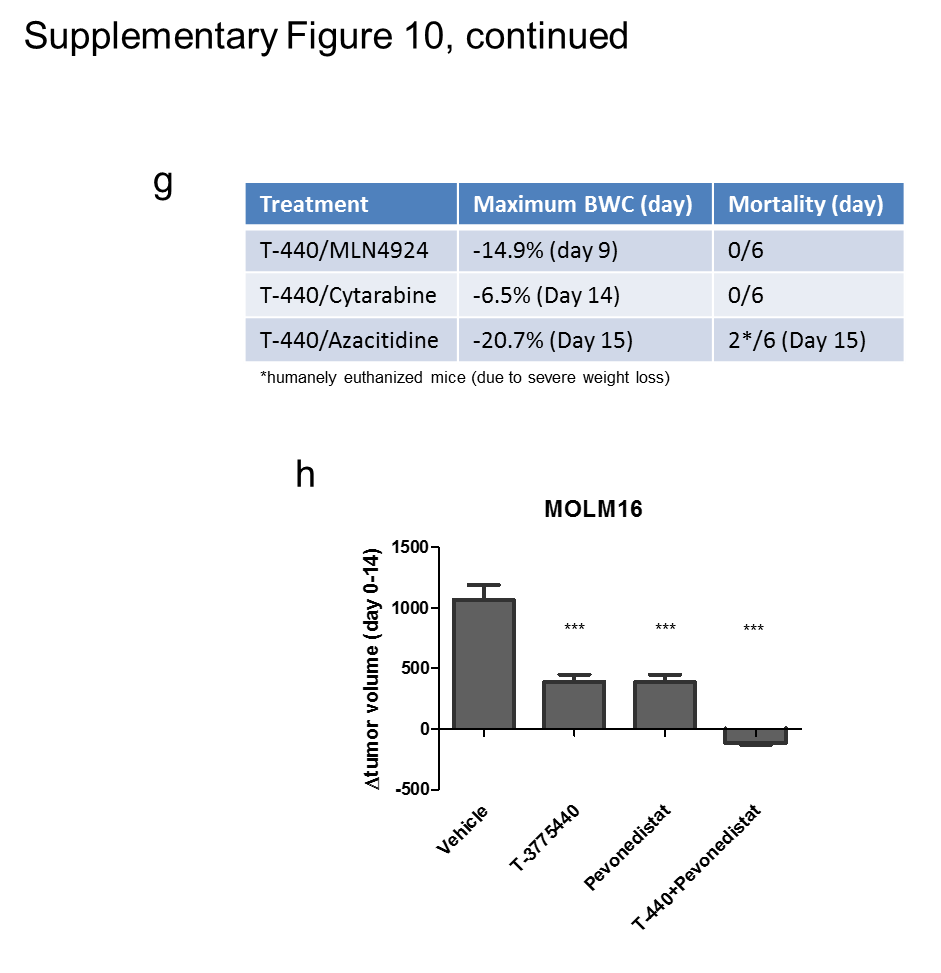


Supplementary Figure 10 (related to Fig. 4). The T-3775440/pevonedistat combination exhibits significant anti-AML effects in subcutaneous xenograft models. (a–g) Antitumor effects of T-3775440 in combination with pevonedistat (a, b), cytarabine (c, d), or azacitidine (e, f) were examined in TF-1a tumor subcutaneous models. Tumor growth curves for each individual mouse (a, c, e) and the changes in tumor volumes (b, d, f) from day 0 to day 15 are represented. The values represent mean tumor volume changes ± SEM (*n* = 5). Statistical significance was determined using Dunnett's multiple comparison test (^*^*P* < 0.05, ^***^*P* < 0.001). Animals received T-3775440 (once daily, on a 5 days on/2 days off schedule, *po*), pevonedistat (three times weekly, on days 1, 3, and 5, *sc*), cytarabine (three times weekly, on days 1, 3, and 5, *ip*), or azacitidine (twice weekly, on days 1 and 4, *sc*). (g) Maximum body weight change (BWC) and mortality rate were represented for each combination. (h) Antitumor effects of T-3775440 in combination with pevonedistat in a MOLM16 tumor subcutaneous model. The changes in tumor volumes from day 0 to day 14 are represented.


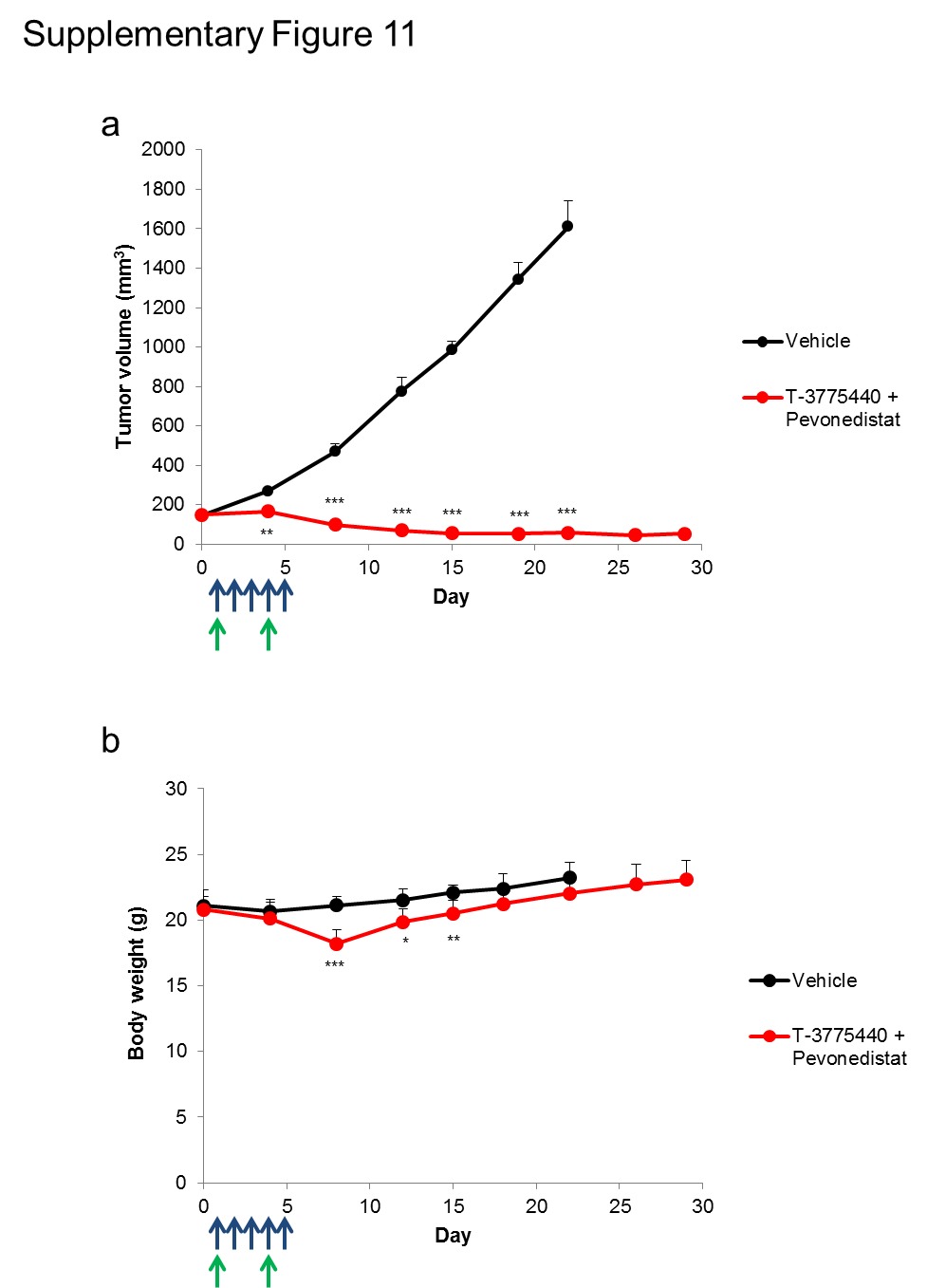


Supplementary Figure 11. One cycle treatment of the T-3775440/pevonedistat combination induced sustained tumor regression. Mice bearing TF-1a tumors were treated with T-3775440 (*po*) once daily for 5 consecutive days and pevonedistat (*sc*) on days 1 and 4 (*n* = 6). (a) *Values*, mean tumor volume; *bars*, SEM. (b) *Values*, mean body weight; *bars*, SD. ^*^*P* < 0.05, ^***^*P* < 0.001. Aspin-Welch t test was used for the statistical analyses.

Supplementary Figure 12. Coadministration of T-3775440/pevonedistat shows anti-leukemic effects in an AML disseminated model. (a) Tumor growth was monitored on days 11, 18, and 25 after *ip* injection with luciferin, using an *in vitro* imaging system. Representative images of mice are shown. (b) Whole body luminescence was measured after the indicated treatment on day 25. Statistical significance was determined using Dunnett's multiple comparison test (^***^*P* < 0.001). *Values*, mean body weight; *bars*, SD. (c) Two mice treated with T-3775440/pevonedistat combination did not show any sign of tumor regrowth (day 133).
